# Arterial cyclic stretch regulates Lamtor1 and promotes neointimal hyperplasia via circSlc8a1/miR-20a-5p axis in vein grafts

**DOI:** 10.7150/thno.69551

**Published:** 2022-06-21

**Authors:** Ji-Ting Liu, Qing-Ping Yao, Yi Chen, Fan Lv, Ze Liu, Han Bao, Yue Han, Ming-Liang Zhang, Zong-Lai Jiang, Ying-Xin Qi

**Affiliations:** 1 Institute of Mechanobiology & Medical Engineering, School of Life Sciences & Biotechnology, Shanghai Jiao Tong University, Shanghai, China.; 2 Department of Pediatric Surgery, Xinhua Hospital Affiliated to Shanghai Jiao Tong University School of Medicine, Shanghai, 200092, China.; 3 Department of Endocrinology and Metabolism, Shanghai Jiao Tong University Affiliated Sixth People's Hospital, Shanghai Diabetes Institute, Shanghai Clinical Center for Diabetes, Shanghai Key Laboratory of Diabetes Mellitus, Shanghai Key Clinical Center for Metabolic Disease, Shanghai, China.

**Keywords:** Lamtor1, circSlc8a1, mTORC1, cyclic stretch, vein graft

## Abstract

**Rationale:** Neointimal hyperplasia caused by dedifferentiation and proliferation of venous smooth muscle cells (SMCs) is the major challenge for restenosis after coronary artery bypass graft. Herein, we investigated the role of Lamtor1 in neointimal formation and the regulatory mechanism of non-coding RNA underlying this process.

**Methods:** Using a “cuff” model, veins were grafted into arterial system and Lamtor1 expression which was correlated with the activation of mTORC1 signaling and dedifferentiation of SMCs, were measured by Western blot. Whole transcriptome deep sequencing (RNA-seq) of the grafted veins combined with bioinformatic analysis identified highly conserved circSlc8a1 and its interaction with miR-20a-5p, which may target *Lamtor1*. CircSlc8a1 was biochemically characterized by Sanger sequencing and resistant to RNase R digestion. The cytoplasmic location of circSlc8a1 was shown by fluorescence *in situ* hybridization (FISH). RNA pull-down, luciferase assays and RNA immunoprecipitation (RIP) with Ago2 assays were used to identify the interaction circSlc8a1 with miR-20a-5p. Furthermore, arterial mechanical stretch (10% elongation) was applied *in vitro*.

**Results:** *In vivo*, Lamtor1 was significantly enhanced in grafted vein and activated mTORC1 signaling to promote dedifferentiation of SMCs. Arterial mechanical stretch (10% elongation) induced circSlc8a1 expression and positively regulated Lamtor1, activated mTORC1 and promoted SMC dedifferentiation and proliferation. Local injection of circSlc8a1 siRNA or SMC-specific *Lamtor1* knockout mice prevented neointimal hyperplasia in vein grafts *in vivo*.

**Conclusions:** Our study reveals a novel mechanobiological mechanism underlying the dedifferentiation and proliferation of venous SMCs in neointimal hyperplasia. CircSlc81/miR-20a-5p/Lamtor1 axis induced by arterial cyclic stretch may be a potential clinical target that attenuates neointimal hyperplasia in grafted vessels.

## Introduction

Coronary artery bypass graft (CABG) surgery is widely used for the treatment of occlusive arterial disease especially for 3-vessel or left main coronary artery disease, and the autologous saphenous vein is still the most frequently used conduit for CABG 1. However, the long-term patency of vein grafts is unsatisfactory, and extensive neointimal hyperplasia leading to accelerated restenosis is the major causes of vein graft failure 2. Growing evidences have proved that the dysfunctions of smooth muscle cells (SMCs) participate in the neointimal formation after vein graft 3.

SMCs are normal cellular components of the medial layer of vessel walls. Aberrant proliferation, migration and dedifferentiation of venous SMCs are the core events in the process of neointimal hyperplasia 3. It has been reported that growth factors and mechanical stresses can change the phenotype of venous SMCs from a quiescent contractile phenotype to a highly proliferative and synthetic phenotype 4. Researches revealed that after a vein grafted into the arterial circulation, venous SMCs are immediately exposed to arterial cyclic stretch caused by rhythmic deformation of the vessel wall following the arterial blood pressure cycle 5. Arterial cyclic stretch can phosphorylate Akt, activate Rho/Rho-kinase and induce the proliferation of human saphenous vein SMCs 6. Mechanically stimulated SMCs underwent transition from contractile to synthetic phenotype which promoted human saphenous vein remodeling 7. An *in vivo* model also revealed that vascular external stent for vein grafts prevents graft failure due to the reduction in wall stretch 8. Although related studies have revealed that arterial mechanical stretch plays important roles in neointimal hyperplasia and vein graft failure, the molecular mechanism is still far from fully understood.

Recent evidences indicate that Lamtor1 (also named p18), a highly conserved late endosome/lysosome membrane adaptor protein, plays an important role in the activation of mammalian target of rapamycin complex 1 (mTORC1), an important modulator in various cardiovascular homeostasis and pathophysiological processes 9. Lamtor1 is a crucial component of the lysosomal adaptor protein complex Ragulator, and its 45-amino acid N-terminal tail contains myristoyl and palmitoyl modifications which is necessary for the localization of Ragulator complex to lysosomes thus directly interacts with mTORC1 10. Lamtor1 allows mTORC1 to localize on the lysosomal surface for activated Rheb GTPase 11. Current studies underscored the pivotal role of Lamtor1 in activating mTORC1 and subsequently regulating cell growth and homeostasis. For example, Lamtor1 knockdown reduces mTORC1 activity and improves dendritic spine maturation and learning performance 12. Ada Nowosad found that CDKN1B promotes starvation-induced autophagic flux and apoptosis by repressing mTORC1 through Lamtor1 13. More interestingly, mTOR signaling is activated by mechanical stretch 14 and primary cilia which is an important mechanosensor 15. However, the mechanism by which mTORC1 responses to mechanical stimuli is still not clear. Here, we explored whether Lamtor1 is mechanoresponsive and then modulate the activation of mTOR signaling, as well as their mechanobiological roles in dedifferentiation and proliferation of venous SMCs in neointimal hyperplasia after vein graft.

In the present study, using a vein graft animal model with the “cuff” technique *in vivo* and FX-5000T strain unit *in vitro*, the effects of arterial cyclic stretch on Lamtor1 expression and venous SMC differentiation were investigated. Whole transcriptome sequencing data combined with bioinformatics analysis hinted that circSlc8a1 may act as a sponge of miR-20a-5p to target on Lamtor1. Based on SMC-specific *Lamtor1* knockout (KO) mice, we further examined whether Lamtor1 and the related noncoding RNA (ncRNAs) may provide novel potential therapeutic targets to prevent neointimal formation and vein graft failure.

## Methods

An expanded Methods section is available in the Supplementary material.

### Animals

Animal experiments were performed conform to the Animal Management Rules of China (Documentation 55, 2001, Ministry of Health, China). The animal studies were approved by the Animal Research Committee of Shanghai Jiao Tong University.

### SMC-specific Lamtor1 KO mice

The *SM22Cre*^+^ mice were purchased from Shanghai Model Organisms Center. For generation of SMC-specific *Lamtor1* KO mice on a C57BL/6J background, *Lamtor1*^fl/fl^ mice were crossed with *SM22Cre*^+^ mice. The generated *Lamtor1*^fl/fl^*SM22Cre*^+^ mice were genotyped by PCR, and littermate wild type (WT) mice were used as controls. The *Lamtor1* KO mice and C57BL/6J background littermate controls were maintained on a light/dark (12/12 h) cycle at 25 °C and received food and water *ad libitum*. Ten- to 12-wk-old male mice were used for further experiments.

### Vein graft animal model

Vein graft was performed using the “cuff” technique 16, 17. In brief, 10- to 12-wk *Lamtor1* KO mice or C57BL/6J background littermate control mice were anesthetized by 2% isoflurane at 1 L/min oxygen flow using an isoflurane vaporizer (Matrx VIP 3000). The inferior vena cava was harvested from a donor mouse. In the recipient mouse, the left common carotid artery was exposed and dissected in the middle. Cuffs (1 ± 0.2 mm, polyimide tubing 141-0021, Nordson Medical, San Jose, CA) were placed on both ends of the divided artery. The vein from the donor mouse was then interposed between the two ends of the carotid artery by sleeving the ends of the vein over the artery cuff and ligating them together with 8-0 silk suture. One wk later, the mice were anesthetized by 2% isoflurane at 1 L/min oxygen flow, and the vein grafts were harvested for further analysis. The inferior vena cava was used as autologous control.

### siRNA treatment

For circSlc8a1 knockdown, circSlc8a1-specific siRNA or the corresponding nonsilencing siRNA control (Shanghai GenePharma Co., Ltd.) was subcutaneously injected around the grafted vein using 1 mL injection syringe with 16G needle *in vivo*. For the first injection, 20 nmol circSlc8a1-specific siRNA or nonsilencing control was subcutaneously injected, and then 15 nmol was administered every day for 1 wk. The efficacy of siRNA treatment was verified by 5'-carboxyfluorescein-labeled siRNA (FAM siRNA) and RT-qPCR.

### Venous SMCs culture

Rat venous SMCs were isolated from jugular vein of male SD rats (150-180 g) as previously described 18. The vein was cut into small pieces and cultured in Dulbeco's Modified Eagle medium (DMEM, Gibco) with 10% fetal bovine serum (FBS, Gibco), in a humidified incubator at 37 °C, 5% CO_2_. The purity of cultured venous SMCs was identified by immunofluorescent staining with α-smooth muscle actin (α-SMA) and purity more than 95% (Figure S1). Venous SMCs from passage 4 to 7 were used for further assays.

### High-throughput sequencing

Total RNA was extracted from grafted vein by Trizol reagent (Invitrogen), and the RNA quality and quantity were checked with Bioanalyzer 2200 (Aligent). The cDNA libraries were constructed using the NEBNext® Ultra™ Directional RNA Library Prep Kit for Illumina according to the manufacturer's instructions. The clean reads were then aligned to rat genome (version: Rnor6 NCBI) using the hisat2 19. Differential gene and transcript expression analysis of RNA-seq experiments with HTseq was used to count mRNA and ncRNA, and FPKM method was used to determine the gene expression 20. DESeq2 algorithm was applied to filter the differentially expressed genes with following criteria: fold change > 2 and false discovery rate (FDR) < 0.05 21.

### Mechanical cyclic stretch application

Venous SMCs were plated on flexible silicone elastomer-bottom plates (Flexcell International Corporation, USA) at a density of 2×10^5^ per well. After 24 h plating, the cells were incubated with DMEM for 24 h for synchronization. Venous SMCs were then subjected to cyclic stretch with FX 5000T (FlexCell International Corporation, USA) as described previously 22. Cyclic stretch with 10% elongation was used to mimic arterial mechanical condition 23 at 1.25 Hz for 6, 12 and 24 h, respectively. The cells under the same conditions but with no mechanical stretch application were used as static control.

Venous SMCs were treated with Rapamycin (mTORC1 inhibitor, 50 nM) after cells were incubated with DMEM for 24 h, and then subjected to cyclic stretch. The vehicle was used as the control.

### RNA pull-down assay

The BersinBio^TM^ RNA pulldown kit (BersinBio, Guangzhou, China) was used to detect the interactions between circSlc8a1 and microRNAs (miRNAs). In brief, the specific biotin-labeled circSlc8a1 probe was denatured at 50 °C for 2 min and incubated with pre-cooled RNA structure buffer. The circSlc8a1 probe has 25 bp oligonucleotide which designed for the specific splice junction site of circSlc8a1 to avoid the interference from linear RNA. Streptavidin magnetic beads (Invitrogen) were then incubated with the mixture at 37 °C for 30 min. The reaction mixture was eluted and RNA was purified by centrifugation at 16100 g for 30 min. The sediments were washed with 80% ethyl alcohol, dissolved in DEPC water, and then analyzed with RT-qPCR. The sequence of circSlc8a1 specific probe was listed in Table S1.

### RNA immunoprecipitation

Briefly, VSMCs (1 × 10^7^) were incubated with lysis buffer including protease and RNase inhibitors. Then the cell lysis was incubated with magnetic beads conjugated with ani-Ago2 antibody (CST) or negative control IgG, at 4 °C overnight. Subsequently, samples were washed and incubated with Proteinase K. Immunoprecipitated RNA was purified and was analyzed with RT-qPCR to determine circSlc8a1 and miR-20a-5p expression.

### Dual luciferase reporter assay

Reporter assays were conducted using the Dual Luciferase Reporter Assay System (Promega) according to the manufacturer's instructions. The 3' untranslated region (3'UTR) of circSlc8a1 including the conserved miR-20a-5p binding sequences or the mutated sequences was obtained by gene synthesis and then inserted into downstream of the luciferase reporter gene (pmirGLO, Promega). To determine the binding reaction between miR-20a-5p and circSlc8a1, HEK-293T cells were transfected with the reporter plasmid or the mutated vectors together with miR-20a-5p mimics or nonsilencing sequence which was used as a mock control using Lipofectamine 2000 (Invitrogen). After 24 h, firefly and *Renilla* luciferase activities were measured consecutively. The sequences of wild type and mutant of circSlc8a1 3'UTR were listed in Table S2.

### Statistics

All experiments were performed with at least three biological replicates, and the data were presented as the mean ± the standard deviation (*SD*). Statistical analysis was performed by GraphPad Prism (version 8.1, GraphPad, San Diego, CA). Student's *t* test was used for comparisons between 2 groups. For multigroup comparisons, the Shapiro-Wilk test was used, and 1-way ANOVA was performed followed by Bonferroni's multiple post hoc *t* tests. *P* < 0.05 was regarded as statistically significant. Throughout the figures, ^✳^, ^ØØ^ and ^ØØØ^ indicated *P* values < 0.05, 0.01 and 0.001, respectively.

## Results

### Vein grafting increases the expression of Lamtor1 and induces neointimal hyperplasia

**Figure 1:**
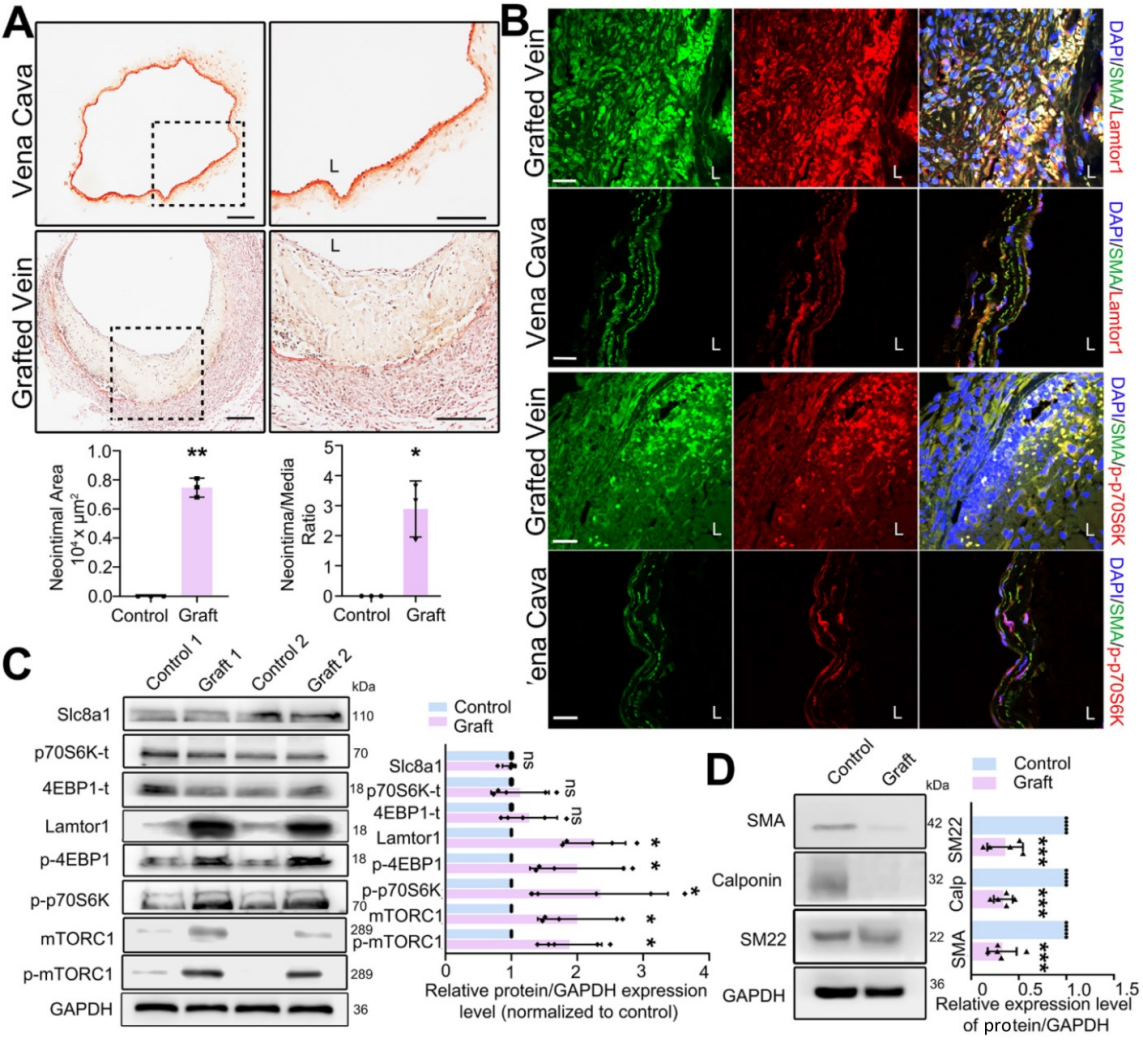
** Lamtor1 expression was increased in grafted vein and positively correlated with neointima hyperplasia and mTORC1 activation *in vivo*. A.** Representative images of Elastin-van Gieson staining, by which neointima is stained yellow and tunica media is red, showed that the neointimal hyperplasia was significantly thickened after 1-wk bypass surgery compared with vena cava (control) in C57BL/6 mice. Image J was used to analyze the neointima to media ratio of the whole vessel. The neointima area and the ratio of the neointima area to the media area were measured and averaged in three independent samples, and each sample from three serial cross-sections. L indicated the vessel lumen. Scale bars: 100 µm. **B.** Immunofluorescence staining of Lamtor1 (red), p-p70SK (red) and smooth muscle α-actin (α-SMA; green) in grafted vein and vena cava (control). Nuclei were counterstained with DAPI (blue). L indicated the vessel lumen. Scale bars: 20 µm. **C.** Protein expressions of Lamtor1, mTORC1, Slc8a1, total 4EBP1 and total p70S6K, as well as phosphorylations of mTORC1, 4EBP1 and p70S6K were analyzed with western blot. The protein expressions in grafted veins were normalized to signals from the autologous jugular vein (control, *n* = 5). **D.** Western blot analyzed the expressions of differentiated markers, including α-SMA, Calponin and SM22, after 1-wk rat grafting surgery (control, *n* = 5). Data were represented as mean ± *SD*. ^✳^*P* < 0.05, ^ØØ^*P* < 0.01, ^ØØØ^*P* < 0.001.

Mouse vein graft model was constructed with the “cuff” technique to mimic clinical bypass surgery. Elastin-van Gieson staining was used to detect the neointima in mouse grafted veins. In the whole vessel, the neointima area was analyzed by Image J, and then normalized to the media area to calculate the intima to media ratio. Compared with that in the vena cava (autologous control), the neointima was significantly thickened after 1-wk grafting (Figure 1A), and the area of neointimal hyperplasia in the grafted vein was markedly increased (Figure 1A). The increased neointimal hyperplasia was also detected in the rat grafted vein by using H&E staining (Figure S2).

To determine the role of Lamtor1 in neointimal formation, we firstly examined the expression of Lamtor1 after vein graft. RNA sequencing and RT-qPCR data revealed that compared with the autologous control, *Lamtor1* was significantly increased in the grafted vein (Table S3 & Figure S3). Additionally, immunofluorescence staining showed that Lamtor1 protein expression was sharply increased in the neointimal area of the grafted vein in both mouse (Figure 1B, Figure S4) and rat model (Figure S5). The increased Lamtor1 was primarily colocalized with α-SMA, which suggested the cytoplasm expression of Lamtor1 in venous SMCs. Furthermore, western blot also revealed that the protein level of Lamtor1 was significantly enhanced in the grafted vein compared with the autologous control (Figure 1C). Notably, the phosphorylation of mTORC1, the most important effector of Lamtor1 and a key regulator of protein translation 24, was significantly increased; interestingly, the expression of mTORC1 was also upregulated. The main substrates of mTORC1, including S6 kinase-1 (S6K1) and eukaryotic translation initiation factor 4E (eIF4E) binding protein-1 (4EBP1) were similarly significantly phosphorylated (Figure 1B-C) in the grafted vein. Whereas, the protein expressions of Slc8a1, total S6K1 and total 4EBP1 were not changed between grafted veins and autologous control (Figure 1C).

Our previous work revealed the abnormal proliferation of venous SMCs in neointimal hyperplasia 22, and here, the phenotypic transformation of venous SMCs, which is a fundamental step for proliferation, was analyzed by western blot. The results indicated that the expressions of α-SMA, calponin and SM22 were all repressed in the vein grafts compared with the autologous vein control (Figure 1D).

These data suggested that after a vein grafted into arterial circulation, Lamtor1 expression was increased which was accompanied with mTORC1 activation, as well as dedifferentiation and proliferation of venous SMCs. These processes may participate in neointimal hyperplasia after vein grafting.

### Noncoding RNAs including CircSlc8a1 are differentially expressed in grafted veins

**Figure 2:**
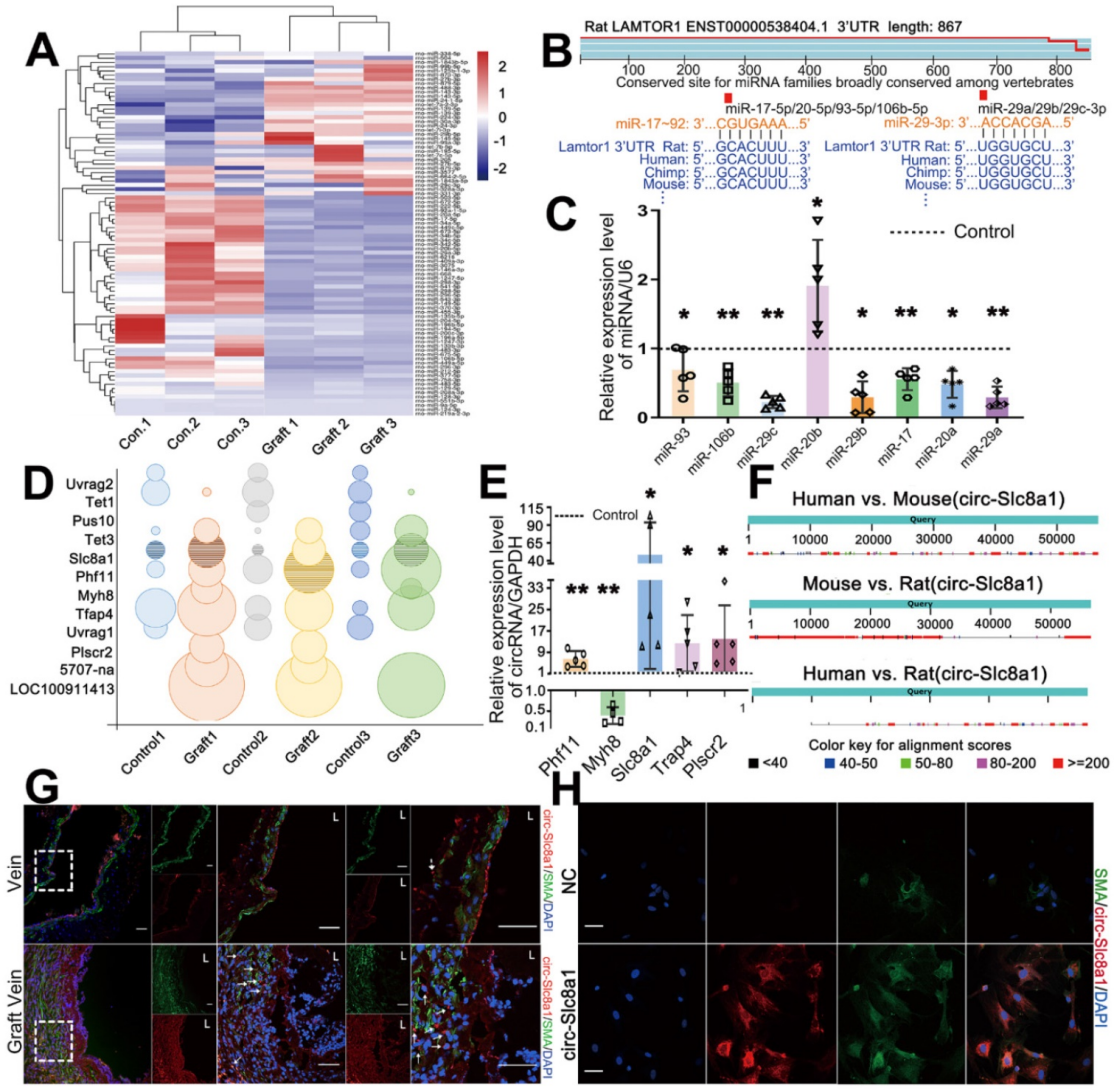
** CircSlc8a1, as a sponge of miR-17-92, promoted Lamtor1 expression. A.** Hierarchical clustering heat map showed the differentially expressed miRNAs, predicted by Miranda package and RNAhybrid that have the binding site with *Lamtor1*, between autologous vein (control) and grafted vein (fold change > 2, FDR < 0.05, *n* = 3). **B.** miR-17-92 cluster and miR-29 cluster, highly conserved across vertebrate species, were predicted to bind with *Lamtor1* 3'UTR. **C.** RT-qPCR detected the expressions of miR-17-92 cluster and miR-29 cluster in autologous vein (control) and grafted vein (*n* = 5). **D.** Bubble chart revealed the differentially expressed circRNAs between autologous vein (control) and grafted vein (fold change > 2, FDR < 0.05, *n* = 3). The grid bubbles represented circSlc8a1. E. RT-qPCR determined circRNA expressions in autologous vein (control) and grafted vein (*n* = 5). **F.** Conservative analysis of circSlc8a1 among human, mouse and rat. **G.** FISH revealed circSlc8a1 expression (red) in autologous vein and grafted vein. α-SMA was stained in green, and nuclei were counterstained with DAPI (blue). Arrows represented the increased circSlc8a1 in tunica media and neointima. L indicated the vessel lumen. Scale bars: 20 µm. **H.** FISH revealed that circSlc8a1 (red) was located in cytoplasm of cultured venous SMCs. α-SMA was stained green, and nuclei were counterstained with DAPI (blue). Scale bars: 20 µm. Data were represented as mean ± *SD*. ^✳^*P* < 0.05, ^ØØ^*P* < 0.01.

To gain further insight into the regulatory mechanism underlying Lamtor1 regulation in vein grafting, RNA-seq of grafted veins and autologous vein controls was performed. Since mounting evidences have demonstrated that ncRNAs are key epigenetic regulators in the cardiovascular diseases 25, we then focused on miRNAs that harbor the ability to post-transcriptionally control mRNA/protein expression. The results showed that there were 86 differentially expressed miRNAs that had predicted binding sites with *Lamtor1,* of which 52 miRNAs were downregulated in the grafted veins compared with the autologous controls (Figure 2A).

According to TargetScan (http://www.targetscan.org/) database, miR-17-92 cluster and miR-29 cluster were highly conserved across vertebrate species, and were predicted to be involved in Lamtor1 regulation (Figure 2B). A miRNA cluster contains two or more miRNAs that acting as a family, because they are frequently highly sequentially homologous especially in the seed region and are transcribed from physically adjacent sequences in the same orientation 25. The predicted seed regions of these two miRNA clusters binding with *Lamtor1* 3'-UTR were shown in Figure 2B. To further validate the differential expressions of miR-17-92 cluster and miR-29 cluster, RT-qPCR was performed. The results indicated significant repressions of the miR-17-92 cluster and miR-29 cluster in grafted veins, except miR-20b-5p (Figure 2C).

RNA-seq also revealed 8 upregulated circRNAs and 4 downregulated circRNAs in the grafted vein compared with the autologous control (Figure 2D). To validate the expressions of these candidate circRNAs, RT-qPCR using splice junction-specific divergent primers was performed. Consistent with RNA-seq results, circSlc8a1, circPhf11, circTrap4 and circPlscr2 were significantly up-regulated in grafted veins, while circMyh8 was down-regulated which was inconsistently. Among these consistent candidates, circSlc8a1 was the most significantly upregulated circRNA in the grafted veins compared to the autologous controls (Figure 2E), and was the only conserved circRNA in humans, mice and rats, as revealed by CircBase (http://www.circbase.org/) (Figure 2F). According to the binding energies of circRNAs and miRNAs, circSlc8a1 had potential binding ability with both miR-17-5p cluster (miR-17-5p and miR-20a-5p) and miR-29 cluster (miR-29a-3p and miR-29c-3p) (Table S4). Hence, the next studies were focused on circSlc8a1.

With a biotin-labeled splice junction-specific probe, fluorescence *in situ* hybridization (FISH) indicated that positive circSlc8a1 staining and SMA positive cells were all mainly expressed in the neointima* in vivo* (Figure 2G, Figure S6). In cultured venous SMCs, circSlc8a1 colocalized with α-SMA in the cytoplasm (Figure 2H) which also suggested a potential miR sponge role.

### Characterization and validation of circSlc8a1 and the role of circSlc8a1 in venous SMC proliferation and dedifferentiation

**Figure 3:**
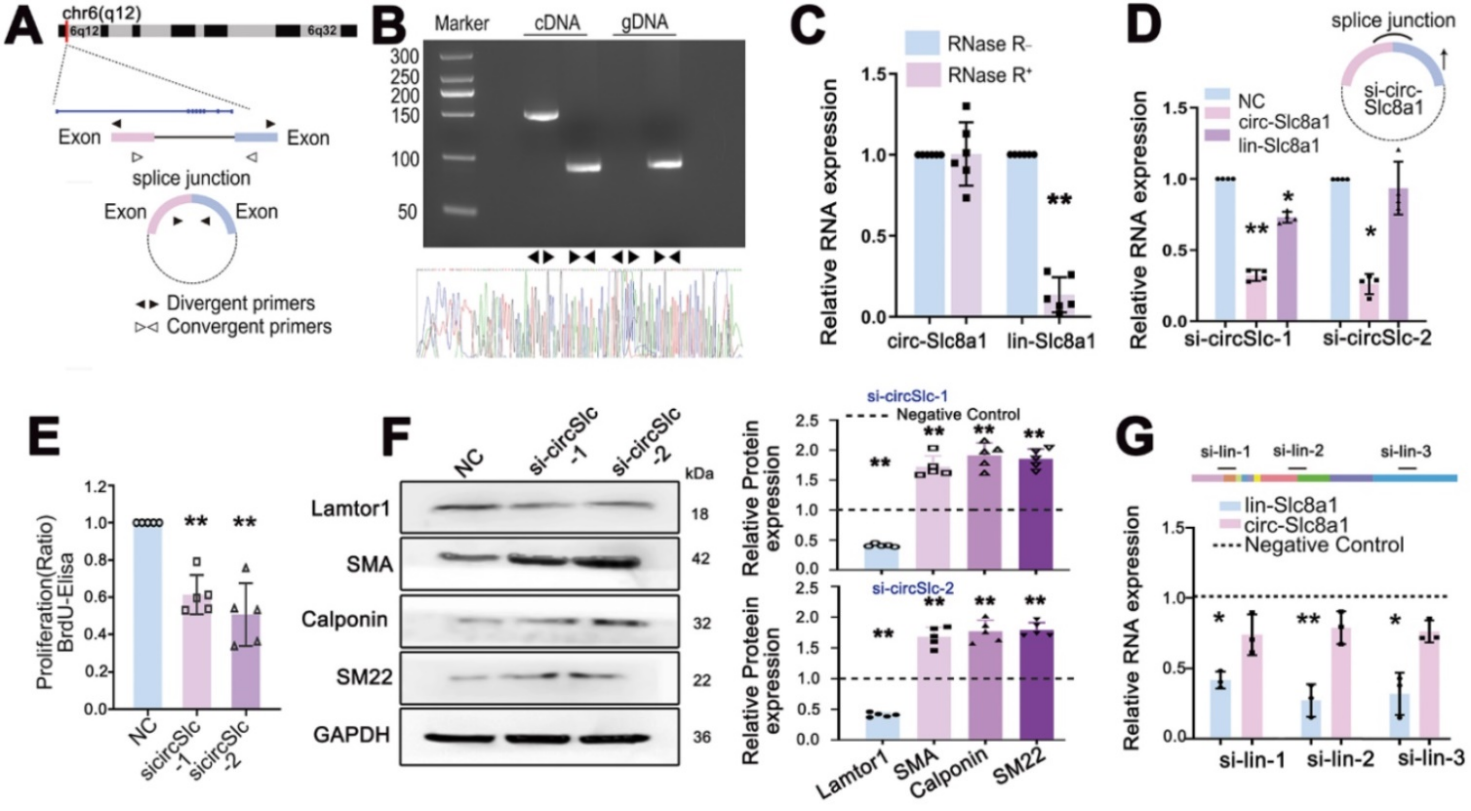
** Characterization and validation of circSlc8a1 in venous SMCs. A.** Schematic illustration showed the circularization of *Slc8a1* exon forming circSlc8a1. **B.** The existence of circSlc8a1 in venous SMCs was validated by RT-qPCR and confirmed by Sanger sequencing. Divergent primers amplified circSlc8a1 in cDNA but not in genomic DNA (gDNA). **C.** After treated with or without RNase R, the expressions of circSlc8a1 and linear *Slc8a1* in venous SMCs were detected by RT-qPCR. The relative RNA levels were normalized to the mock group. RNase R treatment decreased the linear *Slc8a1* more significantly than circSlc8a1 (*n* = 5). **D.** RT-qPCR revealed the expressions of circSlc8a1 and linear *Slc8a1* in venous SMCs after transfection with sicircSlc8a1 or nonsilencing control (NC) (*n* = 4). **E.** BrdU ELISA revealed that cell proliferation was significantly decreased after transfection with circSlc8a1 specific siRNAs or nonsilencing control (NC) (*n* = 5). **F.** Lamtor1 expression was significantly reduced and differentiated markers were increased by circSlc8a1 inhibition as determined by western blot (*n* = 5). G. RT-qPCR revealed the expression of linear *Slc8a1* and circSlc8a1 in venous SMCs after transfection with* Slc8a1* siRNA or negative control (*n* = 3). Data were represented as mean ± *SD*. ^✳^ *P* < 0.05, ^ØØ^ *P* < 0.01.

The head-to-tail splicing structure of circSlc8a1 arising from the linear *Slc8a1* gene was shown in Figure 3A. The back-spliced junction of circSlc8a1 was amplified using divergent primers and confirmed by Sanger sequencing (Figure 3B). To verify that circSlc8a1 was circular but not the product of trans-splicing or genomic rearrangements, cDNA and genomic DNA (gDNA) from venous SMCs were used as templates respectively. CircSlc8a1 was only amplified from cDNA by divergent primers, while no amplification product was observed from gDNA (Figure 3B). Moreover, circSlc8a1 showed a strong resistance to the digestion by RNase R, but the linear *Slc8a1* was susceptible (Figure 3C).

Specific siRNAs, targeting the back-spliced junction of circSlc8a1 were designed to knock down the expression of circSlc8a1 and investigate its role in the proliferation and dedifferentiation of venous SMCs. The results showed that the siRNA specifically repressed the expression of circSlc8a1, but had little effect on the expression of linear *Slc8a1* (Figure 3D). BrdU-ELISA revealed that circSlc8a1 knockdown by using two respective siRNAs decreased the proliferation of venous SMCs (Figure 3E), and the expressions of venous SMC differentiation markers, i.e. α-SMA, calponin and SM22, were significantly increased (Figure 3F). Downregulation of circSlc8a1 also repressed the expression of Lamtor1 (Figure 3F).

To confirm the effect of circSlc8a1, siRNA specifically targeting linear *Slc8a1* was transfected. The repression of linear *Slc8a1* had no effect on circSlc8a1 expression (Figure 3G), Lamtor1 expression and venous SMC dedifferentiation (Figure S7).

### CircSlc8a1 positively regulates Lamtor1 by acting as a sponge for miR-20a-5p in venous SMCs

**Figure 4:**
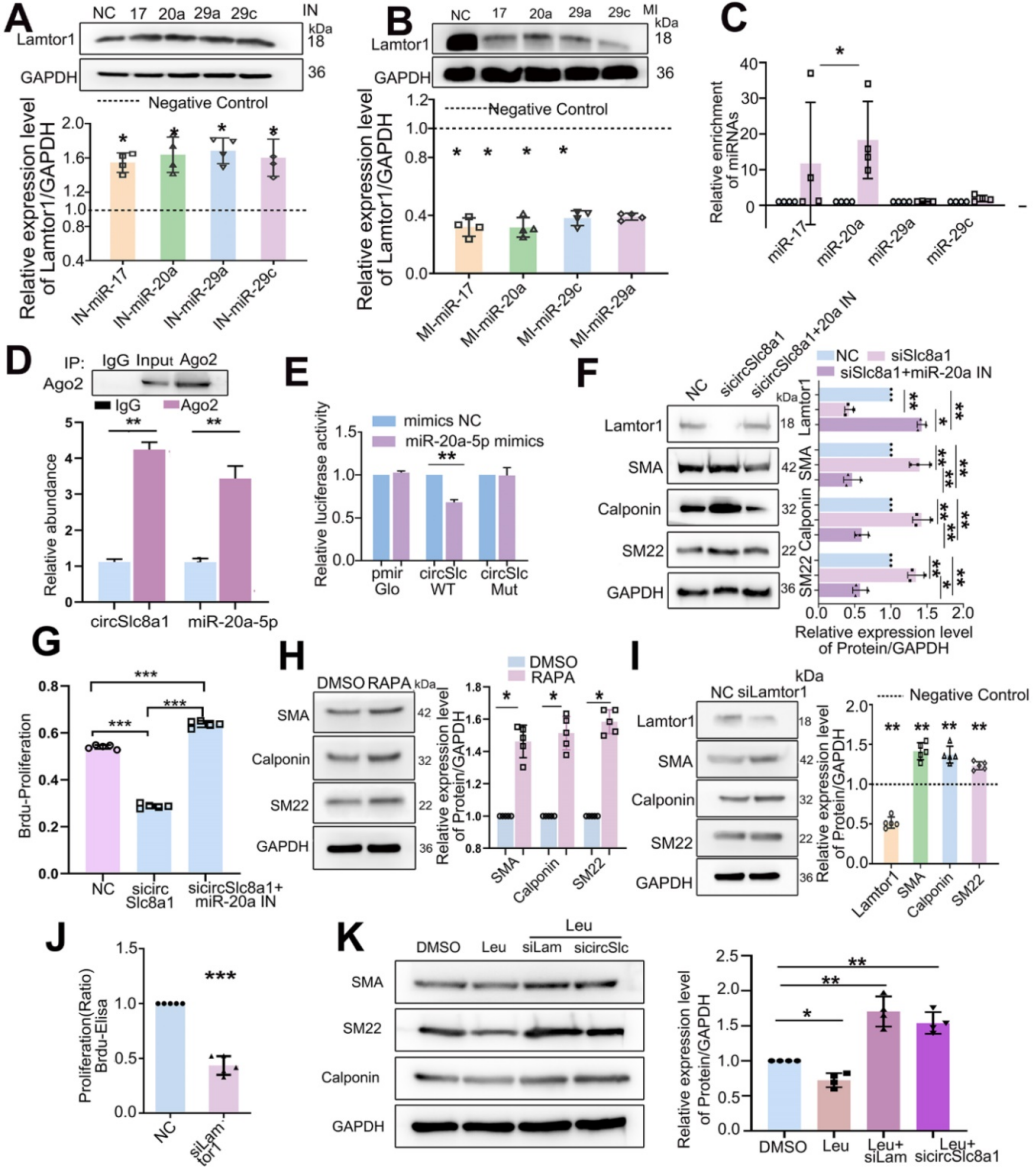
** CircSlc8a1 positively regulated Lamtor1 expression and venous SMC dedifferentiation and proliferation *via* sponging for miR-20a-5p. A.** Western blot indicated that in venous SMCs, inhibitor of miR-17-5p, miR-20a-5p, miR-29a-3p or miR-29c-3p increased Lamtor1 expression compared with the negative control (NC) (*n* = 4). **B.** In venous SMCs, mimics of miR-17-5p, miR-20a-5p, miR-29a-3p or miR-29c-3p decreased Lamtor1 expression compared with NC (*n* = 4). **C.** Among the 4 miRNA candidates, only miR-20a-5p was significantly pulled down with circSlc8a1 biotin-coupled probe in lysates of venous SMCs. **D.** RIP assay was conducted using the Ago2 or IgG antibody for immunoprecipitation. The expressions of circSlc8a1 and miR-20a-5p were detected by RT-qPCR in venous SMCs (*n* = 3). **E.** Luciferase reporter assay with wild type (WT) or mutant (Mut) circSlc8a1 co-transfected with miR-20a-5p mimics or nonsilencing control (NC). Co-tranfection of WT circSlc8a1 and miR-20a-5p significantly reduced luciferase levels (*n* = 3). **F.** The repression of circSlc8a1 inhibited the protein level of Lamtor1, which could be abolished by miR-20a-5p silencing (*n* = 5). **G.** Proliferation of venous SMCs was detected by BrdU-ELISA after transfected with circSlc8a1 specific siRNA (sicircSlc8a1) and simultaneous transfection of miR-20a-5p inhibitor (IN), respectively (*n* = 5). **H.** The expressions of venous SMC differentiation makers were determined by western blot after treated with Rapamycin (50 nM) (*n* = 5). **I.** The expressions of Lamtor1 and differentiation makers were determined by western blot after transfection of* Lamtor1* specific siRNA (*n* = 5). **J.** BrdU-ELISA revealed that specific *Lamtor1* siRNAs decreased venous SMC proliferation compared with nonsilencing control (NC) (*n* = 5). **K.** The expressions of α-SMA, calponin and SM22 were detected by western blot after treated with leucine and the combination with Lamtor1-specific siRNA (siLam) or circSlc8a1-specific siRNA (sicircSlc) (*n* = 5). Data were represented as mean ± *SD*. ^✳^ *P* < 0.05, ^ØØ^ *P* < 0.01, ^ØØØ^ *P* < 0.001.

Since miR-17-5p, miR-20a-5p, miR-29a-3p and miR-29c-3p were predicted to target on *Lamtor1*, respective mimics or inhibitors were transfected and the results showed that the inhibitor of miR-17-5p, miR-20a-5p, miR-29a-3p or miR-29c-3p increased the protein expression of Lamtor1 (Figure 4A), whereas their mimics decreased it (Figure 4B).

A 5'biotin-labeled probe targeting on the splicing junction of circSlc8a1 was designed to determine the potential miRNAs binding with circSlc8a1. RT-qPCR revealed that miR-20a-5p was pulled down by circSlc8a1 specific probe in venous SMCs, while miR-17-5p was also pulled down by circSlc8a1 probe but had no sufficient quantity, and miR-29a-3p and miR-29c-3p revealed no significant pull-down effect (Figure 4C). Anti-Ago2 immunoprecipitation combined with RT-qPCR showed that circSlc8a1 and miR-20a-5p were significantly enriched in the complex precipitated by Ago2 (Figure 4D). To further confirm the binding between miR-20a-5p and circSlc8a1, dual-luciferase reporter assay was performed by constructing WT and mutant (MUT) dual-luciferase reporter vectors of circSlc8a1 according to the potential binding sites with miR-20a-5p. The luciferase activity was significantly decreased by co-transfection of the WT reporter and miR-20a-5p mimics, but not the MUT reporter (Figure 4E). Western blot analysis indicated that circSlc8a1 knockdown repressed Lamtor1 expression and promoted venous SMC differentiation, whereas simultaneous transfection of miR-20a-5p reversed these effects (Figure 4F). Moreover, miR-20a-5p silencing abolished the repressive effect of circSlc8a1 on the Lamtor1 protein expression (Figure 4F). BrdU results showed that circSlca81 specific siRNA inhibited the proliferation of SMCs, while miR-20a-5p repression abolished the repressive effect of circSlc8a blockage (Figure 4G).

Furthermore, we determined whether mTORC1, the effector of Lamtor1, contributed to venous SMC dedifferentiation. Rapamycin, an mTORC1-specific inhibitor, induced venous SMC differentiation (Figure 4H), and Lamtor 1 specific siRNA increased the expressions of contractile markers, including α-SMA, calponin, and SM22 (Figure 4I), as well as suppressed the proliferation (Figure 4J). To verify whether Lamtor1 or cirSlc8a1 were involved in SMC dedifferentiation through mTORC1, the mTORC1 was activated with leucine. Western blot revealed that the expressions of contractile markers, including α-SMA, calponin and SM22, were decreased during mTORC1 activation, while the simultaneous knockdown of Lamtor1 or circSlc8a1 increased the expressions of α-SMA, calponin and SM22 (Figure 4K).

These results validated the direct interaction between circSlc8a1 and miR-20a-5p in venous SMCs. Taken together, the present results showed that circSlc8a1 acts as a miR-20a-5p sponge and positively regulates Lamtor1 expression in venous SMCs.

### Arterial cyclic stretch induces the expression of circSlc8a1 which subsequently increases Lamtor1 expression and SMC dedifferentiation

Once a saphenous vein is transplanted into the arterial system after CABG, the grafting is exposed to arterial mechanical environment including the increased cyclic stretch. We then investigated whether mechanical cyclic stretch modulated the expression of circSlc8a1/miR-20a-5p/Lamtor1 in veinous SMCs.

**Figure 5:**
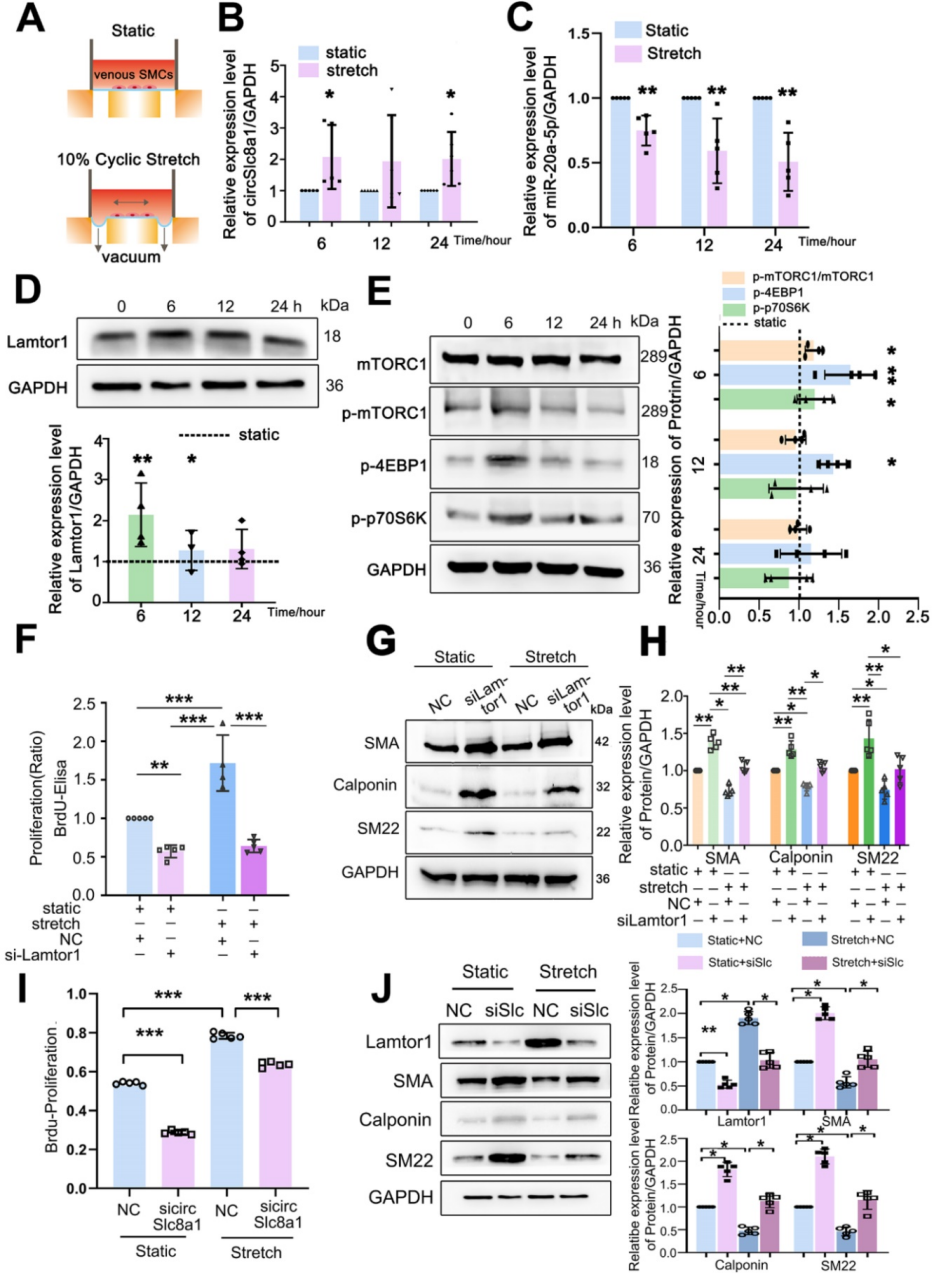
** Arterial mechanical stretch upregulated circSlc8a1 and Lamtor1. A.** Schematic illustration of cyclic stretch-loading system. **B.** CircSlc8a1 expression was significantly increased after applied to 10%-1.25Hz cyclic stretch for 6 and 12 h, respectively (normalized to GAPDH) (*n* = 5). **C.** miRNA-20a-5p expression (normalized to U6) was decreased after 10%-1.25Hz cyclic stretch applied for 6, 12 and respectively (*n* = 5). **D.** Lamtor1 expression was detected by western blot after subjected to 10%-1.25Hz cyclic stretch for 3, 6, 12 and 24 h respectively (*n* = 5). **E.** The expression of mTORC1, as well as the phosphorylations of mTORC1, p70S6k and 4EBP1 were detected by western blot after subjected to 10%-1.25Hz cyclic stretch for 6, 12 and 24 h respectively (*n* = 5).** F.** Proliferation of venous SMCs was detected by BrdU-ELISA after transfected with *Lamtor1*-specific siRNAs under cyclic stretch (*n* = 5). The values of nonsilencing control (NC) transfection were standardized to 1. (**G, H**). Under cyclic stretch, the expressions of α-SMA, calponin and SM22 were detected by western blot after transfected with* Lamtor1*-specific siRNA (*n* = 5). **I.** Proliferation of venous SMCs was detected by BrdU-ELISA after transfected with circSlc8a1-specific siRNAs (sicircSlc8a1) and nonsilencing control (NC) under static and cyclic stretch, respectively (*n* = 5). **J.** The expressions of Lamtor1, α-SMA, calponin and SM22 were detected by western blot after transfected with circSlc8a1-specific siRNA (sicircSlc8a1) and nonsilencing control (NC) under static and cyclic stretch, respectively (*n* = 5). Data were represented as mean ±* SD*. ^✳^ *P* < 0.05, ^ØØ^ *P* < 0.01, ^ØØØ^ *P* < 0.001.

Venous SMCs were subjected to 10%-1.25 Hz cyclic stretch *in vitro* which mimics arterial mechanical stretch *in vivo* (Figure 5A). Compared with the static control, 10% cyclic stretch significantly increased circSlc8a1 expression (Figure 5B), but decreased miR-20a-5p expression (Figure 5C) at 6 h and sustained to 24 h.

The expression of Lamtor1 and the activity of mTORC1 signaling were further examined. 10%-1.25 Hz mechanical stretch significantly upregulated Lamtor1 expression at 6 and 12 h points (Figure 5D), and stimulated the phosphorylation of mTORC1, 4EBP1 and p70S6K (Figure 5E) compared with those under static conditions.

Then we detected whether Lamtor1 participated in venous SMC dedifferentiation and proliferation in response to mechanical cyclic stretch. Mechanical stretch induced 1.7-fold BrdU incorporation compared with the static control (Figure 5F), and decreased the expressions of α-SMA, calponin and SM22, the venous SMC contractile markers (Figure 5G, Figure S8). Whereas *Lamtor1* siRNA reversed the effect of mechanical stretch on venous SMC proliferation (Figure 5F) and dedifferentiation (Figure 5G, H), which was similar to rapamycin treatment under cyclic stretch (Figure S9).

Furthermore, we detected whether cyclic stretch regulated Lamtor1 expression and promoted venous proliferation and dedifferentiation *via* circSlc8a1. The increased proliferation (Figure 5I) and decreased expressions of contractile markers (Figure 5J) induced by mechanical stretch were reversed by the knockdown of circSlc8a1 using siRNA. Meanwhile, circSlc8a1 knockdown reversed the increased Lamtor1 expression in venous SMCs induced by mechanical stretch (Figure 5J).

These results suggested that arterial mechanical stretch induced the expressions of circSlc8a1 and the subsequent Lamtor1, which participated in the dedifferentiation and proliferation of venous SMCs, and may be involved in vascular remodeling during vein graft.

### Downregulated circSlc8a1 suppresses neointima formation and inactivates mTORC1 after vein graft *in vivo*

**Figure 6:**
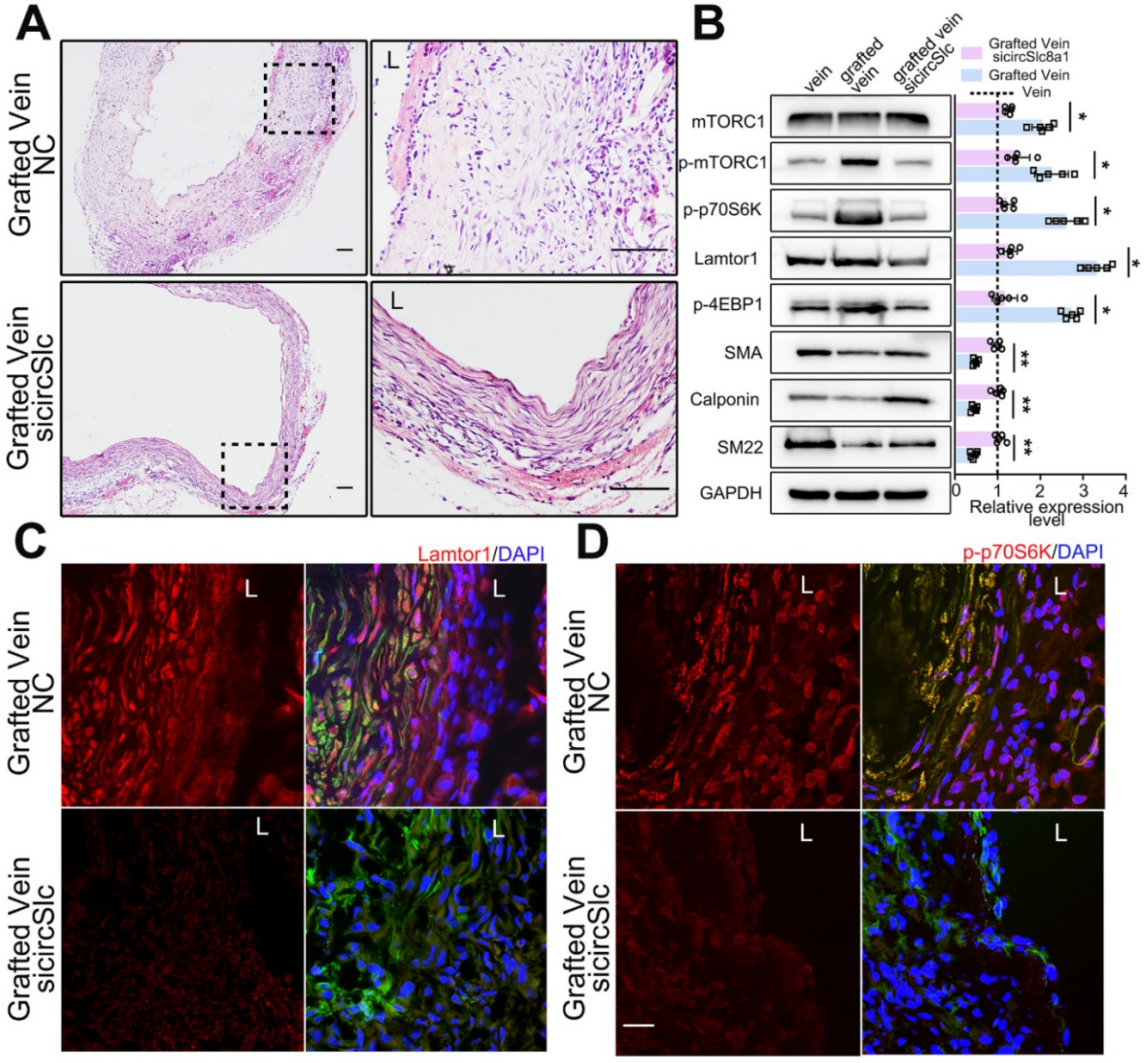
** Down-regulated circSlc8a1 suppressed neointimal formation and mTORC1 activation** ***in vivo*****. A.** Representative H&E images of grafted vein after circSlc8a1-specific siRNA treatment for 1 wk compared with the nonsilencing control (NC). L indicated the vessel lumen. Scale bars: 100 µm. **B.** Western blot detected the expressions of Lamtor1, mTORC1, α-SMA and Calponin, as well as the phosphorylations of mTORC1, 4EBP1 and p70S6K in grafted vein after circSlc8a1-specific siRNA treatment for 1 wk (*n* = 5). Densitometric analysis of protein expression was normalized to signals from the autologous jugular vein (set to 1, black line). **C.** After circSlc8a1-specific siRNA treatment for 1 wk, Lamtor1 (red) and α-SMA (green) were immunofluorescence co-stained in grafted vein. Nuclei were counterstained with DAPI. L indicated the vessel lumen. Scale bars: 20 µm. **D.** After circSlc8a1-specific siRNA treatment for 1 wk, p-p70S6K (red) and α-SMA (green) were immunofluorescence co-stained in grafted vein. Nuclei were counterstained with DAPI. L indicated the vessel lumen. Scale bars: 20 µm. Data represent as mean ±* SD*. ^✳^ *P* < 0.05, ^ØØ^ *P* < 0.01.

After vein graft, specific siRNA targeting circSlc8a1 was administered *via* multipoint subcutaneous injection around the grafted vein to identify the potential role of circSlc8a1 in neointimal formation* in vivo*. The 5'-carboxyfluorescein-labeled siRNA (FAM siRNA) was used to confirm the delivery efficiency of siRNA into the neointima of the grafted vein (Figure S10). CircSlc8a1 expression was significantly decreased in grafted veins treated with specific circSlc8a1 siRNA (Figure S11). CircSlc8a1 knockdown markedly attenuated neointima formation in grafted veins (Figure 6A, Figure S12). Western blot and immunostaining showed that the protein level of Lamtor1 was reduced in the grafted veins in circSlc8a1 knockdown group compared with the negative control (Figure 6B-C, Figure S13). Consistently, western blot revealed that circSlc8a1 knockdown also markedly attenuated the phosphorylation of mTORC1 and the substrates including p70S6K and 4EBP1, and promoted venous SMC differentiation (Figure 6B). Immunostaining confirmed the effect of circSlc8a1 knockdown on p70S6K phosphorylation (Figure 6D, Figure S13).

### SMC-specific deletion of Lamtor1 attenuates neointima formation and inhibits mTORC1 activation after vein graft *in vivo*

**Figure 7:**
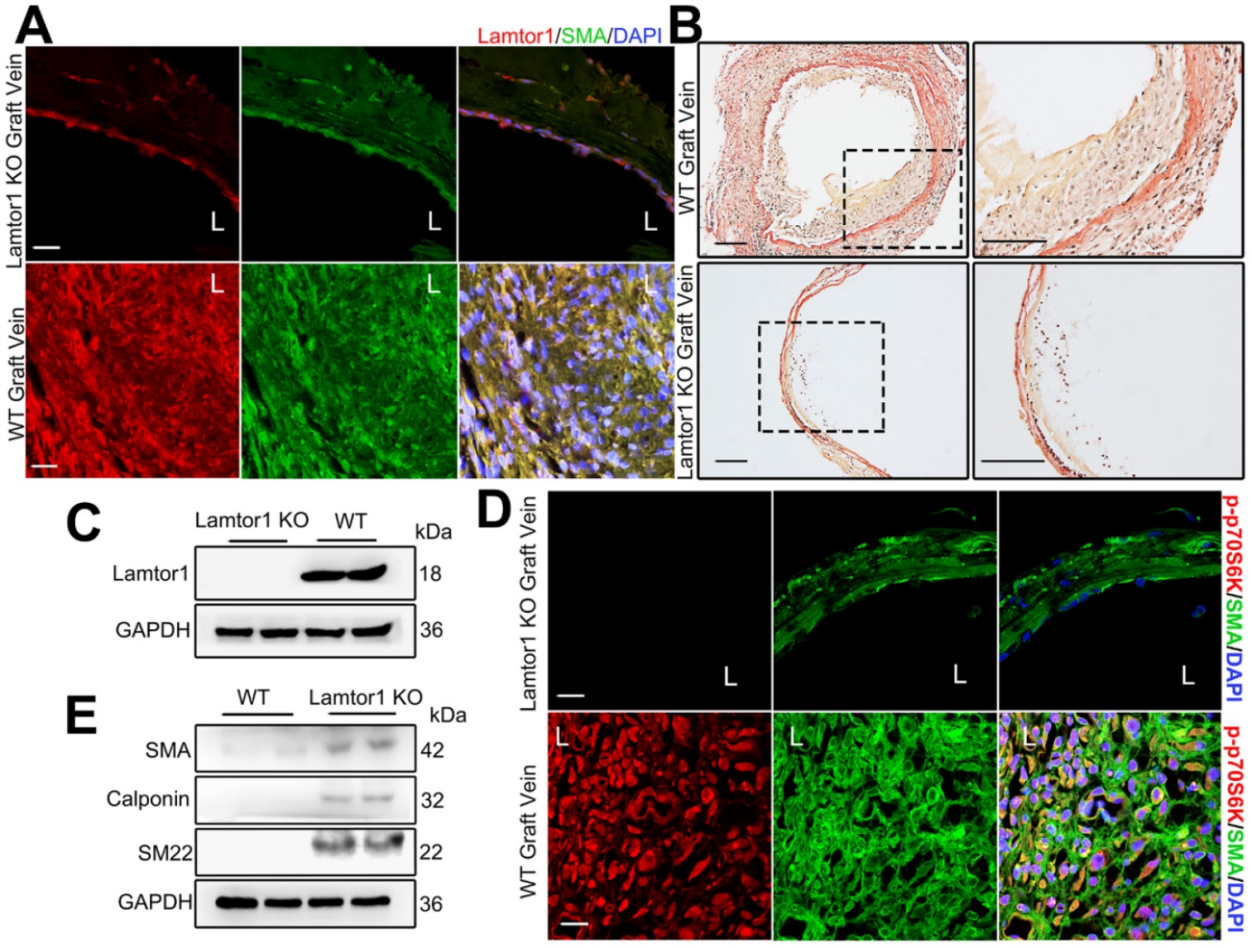
** Ablation of Lamtor1 in SMCs attenuated neointimal formation induced by vein graft surgery. A.** Representative images showed double immunostaining of Lamtor1 (red) and α-SMA (green) in grafted vein of WT and SMC-specific *Lamtor1* KO mice after 1-wk surgery. Nuclei were counterstained with DAPI (blue). L indicated the vessel lumen. Scale bars: 20 µm. **B.** Representative images of Elastin-Van Gieson staining of grafted vein from WT and SMC-specific *Lamtor1* KO mice after 1-wk surgery. Scale bars: 100 µm. **C.** Western blot showed Lamtor1 expression in WT and SMC-specific *Lamtor1* KO mice. **D.** Representative images showed double immunostaining of p-p70S6K (red) and α-SMA (green) in grafted vein of WT and SMC-specific *Lamtor1* KO mice after 1-wk surgery. Nuclei were counterstained with DAPI (blue). L indicated the vessel lumen. Scale bars: 20 µm. **E.** Western blot showed expressions of α-SMA, Calponin and SM22 in grafted vein of WT and SMC-specific *Lamtor1* KO mice after 1-wk surgery.

To assess the role of Lamtor1 in neointimal formation in the grafted vein, we generated SMC-specific *Lamtor1* KO mice. Genomic PCR confirmed the presence of the *SM22Cre* gene in the KO mice compared with the littermate control (Figure S14-15). Immunostaining and western blot revealed that the expression of Lamtor1 was abolished in the media layer of veins of SMC-specific *Lamtor1* KO mice (Figure 7A&C, Figure S16). SMC-specific *Lamtor1* KO displayed a marked reduction of the intimal area and the intima to media ratio in 1-wk vein grafts compared with those of the WT mice (Figure 7B, Figure S17). Next, we investigated whether the specific deletion of Lamtor1 in SMCs suppressed mTORC1 activity *in vivo*. Immunostaining revealed that in SMC-specific *Lamtor1* KO mice p70S6K phosphorylation in vein grafting was significantly decreased when compared with WT (Figure 7D, Figure S16). Furthermore, expressions of contractile proteins, including α-SMA, calponin and SM22, were elevated in the grafted vein of SMC-specific *Lamtor1* KO mice (Figure 7E).

These data suggested that specific KO of Lamtor1 in SMCs *in vivo* attenuated mTORC1 signaling activation and venous SMC dedifferentiation, which also contributed to neointima formation after bypass surgery.

## Discussion

After autogenous bypass graft, the vein undergoes a series of dynamic structural changes to adapt to the arterial hemodynamic forces 26. Physiological saphenous vein is exposed to low pressure (estimated to be 0-3 mmHg) and non-pulsating flow *in vivo*, while after bypass surgery, the mechanical situation changes to high pressure (80-120 mmHg) and pulsating flow 5, 27. The increased cyclic stretch caused by the arterial hemodynamic environment has been shown to play a promoting role in intimal hyperplasia 28. Herein, using *in vivo* vein graft animal model and *in vitro* cyclic stretch application system, we revealed a novel mechanoresponsive pathway, circSlc8a1/miR-20a-5p/Lamtor1, which contributes to the dedifferentiation and proliferation of venous SMCs during neointimal hyperplasia.

After saphenous vein grafting, appropriate vascular remodeling in the grafted vein is thought to be an important component of successful adaptation. However, abnormal or uncontrolled neointimal hyperplasia leads to clinical vein graft failure. Some *in vivo* studies found a stronger dedifferentiation pattern of venous SMCs few months after vein graft 29, 30. SMCs in the dedifferentiation state may subsequently undergo rapid proliferation and migrate from the medial layer into the layer subjacent to the endothelium to form neointimal lesions 3. Interestingly, *in vitro* cultured venous SMCs exhibit a more dedifferentiated phenotype than arterial SMCs, exhibiting heightened proliferative, migratory and synthetic capacities compared to arterial SMCs 31. Therefore, understanding the venous-specific pathophysiological and molecular mechanisms of intimal hyperplasia is important for clinical management after vein grafting.

In animal models, several signaling cascades, including the mitogen-activating protein kinase (MAPK) pathway and phosphatidylinositol 3 kinase (PI3K)-Akt/PKB pathway, have been proved to participate in the proliferation, survival, differentiation and migration of venous SMCs, and the subsequent neointimal formation in grafted vein 32. However, the molecular mechanism in this process is complex and still unclear. Previous studies demonstrated that mTORC1 is closely correlated with cell proliferation and neointima formation 33. Our current study revealed that Lamtor1, as a crucial positive regulator of mTROC1 activation, contribute to the neointima in both rat and mouse vein grafts.

Lamtor1, a lysosome-anchored protein critical for amino acid sensing, is first reported in 2009, and the amino acid sequence is highly conserved in vertebrates 9. Nada et al. reported the ubiquitous expression of Lamtor1 in various organs such as heart, brain, spleen, lung, and muscle 33. Increasing studies have revealed that Lamtor1 is required for cell proliferation. For example, Lamtor1-knockdown in SH-EP cells and HCT116 cells as well as Lamtor1 knockout in splenic CD4+ T lymphocytes 34 and MEFs 35, 36 consistently resulted in decreased proliferation. Our present work suggested that the specific deletion of Lamtor1 in venous SMCs suppresses intimal hyperplasia in grafted vein. Additionally, we demonstrated that deletion of Lamtor1 in SMCs suppresses dedifferentiation, thereby maintaining venous SMCs in a contractile state, and reported the contribution of ncRNAs in this process.

With the advent of next-generation sequencing, numerous circRNAs that represent a large group of heterogeneous ncRNA transcripts have been identified. CircRNAs are derived from pre-mRNAs and are characterized by a closed continuous back spliced loop structure that lacks 5' to 3' polarity 37. Because of the lack of an open-end structure, circRNAs are resistant to exonuclease RNase R digestion and have a longer half-life than linear RNAs 38. Recent studies have revealed that circRNAs are important modulators in a number of cardiovascular diseases. Heart-related circRNA (HRCR) acts as an endogenous miR-223 sponge to inhibit cardiac hypertrophy and heart failure 39. The atheroprotective circANRIL regulates ribosome biogenesis in vascular SMCs and macrophages in human atherosclerotic plaques 40. Based on whole transcriptome sequencing, we focused on circSlc8a1 which was high stability and abundant in organisms, more important it was most upregulated in grafted veins and highly conserved among humans, mice and rats. Wilson L. et al. showed that circSlc8a1 was not only abundant in myocardial cells but also highly expressed in skeletal muscle, liver, stomach, and temporal lobe 41. Knockdown of circSlc8a1 attenuates cardiac hypertrophy from pressure overload, whereas cardiomyocyte-specific overexpression of circSlc8a1 results in heart failure 41. In our present study, we investigated the effects of circSlc8a1 on venous SMC proliferation in response to cyclic stretch. Using JASPAR (https://jaspar.genereg.net/) database, we detected the potential transcription factor regulated *Slc8a1* expression. Among the top 10 transcription factor (Table S5), ZNF384 42, TBX15 43 and CEBPD 44 have been reported to be mechanoresponsive. More interestingly, our recent research revealed that cyclic stretch induces the expression of Serine/arginine-rich splicing factor 1 (SRSF1), a pre-RNA splicing modulator. Whether arterial stretch regulates the back splicing of circSlc8a1 also need future investigation. Together, these studies indicated that circSlc8a1 is mechanoresponsive and is important in the homeostatic and remodeling cardiovascular system.

Herein, using RNA-seq and bioinformatic analysis, 379 differentially expressed miRNAs, 167 differentially expressed lncRNAs, and 12 differentially expressed circRNAs were revealed in grafted veins compared with autologous veins. Among these ncRNS, 34 may participate in cellular dedifferentiation revealed by IPA (Table S6). Interestingly, some of these ncRNAs had been proved to be VSMC differentiation, such as miR-143/145 45, miR-22 46 and et al. We showed a critical role of miR-20a-5p, a member of the miR-17-92 cluster and is highly conserved across vertebrates, in venous SMC differentiation in responsive to mechanical stretch.

The miRNA cluster is a polycistronic group of functionally related miRNAs that contain the same seed sequence 47. The genomic organization of the miRNA cluster is often highly conserved, suggesting important roles in coordinated regulation and function. The miR-17-92 cluster, which sequences are highly conserved in all vertebrates, promotes both cell proliferation and cell survival during normal development 48. MiR-17-92 is one of the most potent oncogenic miRNAs, and an increased expression was observed in a large cohort of human C-cell lymphomas and multiple solid tumor 49. The oncogenic potential of miR-17-92 cluster is firstly shown in a Myc-induced B-cell lymphoma model 50. Overexpression of miR-17-92 cluster in lymphoid lineage cells induces their hyperproliferation leading to lympho-proliferative disease and autoimmunity 51. In neuroblastomas, miR-17-92 cluster shows oncogenic activity and attenuates the TGF-β signaling pathway by repressing the expression of SMAD2, SMAD4 and TGF-β receptor 2 52. In recent years, an increasing number of studies have highlighted the role of miR-17-92 cluster in cardiovascular development and diseases. The proliferation of endothelial cells in the coronary artery is supported by high levels of miR-17-92 cluster, and the loss of endothelial miR-17-92 cluster leads to vascular impairment 53. Moreover, miR-17-92 cluster is regulated by shear stress. The study showed that miR-92a is downregulated by laminar shear stress and upregulates by oscillatory shear stress which subsequently targets on KLF2 and KLF4 and induces endothelial inflammation 54. Our study showed that miR-20a-5p, induced by arterial mechanical stretch, targets on Lamtor1 and inhibits dedifferentiation and proliferation of venous SMCs. Furthermore, circRNAs are endogenous RNAs with having many binding sites distributed throughout their entire sequence. The mechanism by which circRNAs bind with different member of miRNAs in one cluster is still unknown.

In conclusion, our study demonstrates that Lamtor1 induces neointimal hyperplasia after vein graft by activating the mTORC1 signaling pathway and inducing dedifferentiated and proliferation of venous SMCs. CircSlc8a1, in response to arterial cyclic stretch, acts as a sponge for miR-20a-5p and subsequently regulates the expression of Lamtor1. Knockdown of Lamtor1 or circSlc8a1 attenuates neointimal hyperplasia in vein grafts. Therefore, these mechano-responsive molecules including circSlc8a1 and Lamtor1 may provide potential therapeutic targets for neointimal hyperplasia after human autologous vein graft surgery. Although based on the high homology and conservatism, researches on animal models, both *in vivo* and *in vitro*, have provided important suggestions on diagnosis and therapeutic of neointimal formation after CABG surgery, the clinical experiments on circSlc8a1/miR-20a-5p axis are still crucial in the further studies.

## Supplementary Material

Supplementary methods, figures and tables.

## Abbreviations

SMCs: smooth muscle cells
CABG: coronary artery bypass graft surgery
mTORC1: mammalian target of rapamycin raptor complex 1
FISH: fluorescence *in situ* hybridization
ncRNA: noncoding RNA
FBS: fetal bovine serum
DMEM: Dulbeco's Modified Eagle medium
H&E: Hematoxylin/Eosin staining
siRNAs: small interfering RNA
WGBS: whole genome bisulfite sequencing
miRNAs: microRNAs

## References

[1] Puzziferri N, Roshek TB 3rd, Mayo HG, Gallagher R, Belle SH, Livingston EH (2014). Long-term follow-up after bariatric surgery: a systematic review. *JAMA*.

[2] Bulkley BH, Hutchins GM (1977). Accelerated "atherosclerosis". A morphologic study of 97 saphenous vein coronary artery bypass grafts. *Circulation*.

[3] Kenagy RD, Fukai N, Min SK, Jalikis F, Kohler TR, Clowes AW (2009). Proliferative capacity of vein graft smooth muscle cells and fibroblasts *in vitro* correlates with graft stenosis. *J Vasc Surg*.

[4] Shi N, Chen SY (2014). Mechanisms simultaneously regulate smooth muscle proliferation and differentiation. *J Biomed Res*.

[5] Owens CD (2010). Adaptive changes in autogenous vein grafts for arterial reconstruction: clinical implications. *J Vasc Surg*.

[6] Kozai T, Eto M, Yang Z, Shimokawa H, Lüscher TF (2005). Statins prevent pulsatile stretch-induced proliferation of human saphenous vein smooth muscle cells via inhibition of Rho/Rho-kinase pathway. *Cardiovasc Res*.

[7] Garoffolo G, Ruiter MS, Piola M, Brioschi M, Thomas AC, Agrifoglio M (2020). Coronary artery mechanics induces human saphenous vein remodelling via recruitment of adventitial myofibroblast-like cells mediated by Thrombospondin-1. *Theranostics*.

[8] Liu SQ, Ruan YY, Tang D, Li YC, Goldman J, Zhong L (2002). A possible role of initial cell death due to mechanical stretch in the regulation of subsequent cell proliferation in experimental vein grafts. *Biomech Model Mechanobiol*.

[9] Mu Z, Wang L, Deng W, Wang J, Wu G (2017). Structural insight into the Ragulator complex which anchors mTORC1 to the lysosomal membrane. *Cell Discov*.

[10] Sun J, Liu Y, Jia Y, Hao X, Lin WJ, Tran J (2018). UBE3A-mediated p18/LAMTOR1 ubiquitination and degradation regulate mTORC1 activity and synaptic plasticity. *Elife*.

[11] Bar-Peled L, Schweitzer LD, Zoncu R, Sabatini DM (2012). Ragulator is a GEF for the rag GTPases that signal amino acid levels to mTORC1. *Cell*.

[12] Rogala KB, Gu X, Kedir JF, Abu-Remaileh M, Bianchi LF, Bottino AMS (2019). Structural basis for the docking of mTORC1 on the lysosomal surface. *Science*.

[13] Nowosad A, Jeannot P, Callot C, Creff J, Perchey RT, Joffre C (2020). p27 controls Ragulator and mTOR activity in amino acid-deprived cells to regulate the autophagy-lysosomal pathway and coordinate cell cycle and cell growth. *Nat Cell Biol*.

[14] Cheng J, Wang Y, Ma Y, Chan BT, Yang M, Liang A (2010). The mechanical stress-activated serum-, glucocorticoid-regulated kinase 1 contributes to neointima formation in vein grafts. *Circ Res*.

[15] Wiczer BM, Kalender A, Thomas G (2010). Bending the path to TOR. *Nat Cell Biol*.

[16] Bongaarts A, van Scheppingen J, Korotkov A, Mijnsbergen C, Anink JJ, Jansen FE (2020). The coding and non-coding transcriptional landscape of subependymal giant cell astrocytomas. *Brain*.

[17] Zou Y, Dietrich H, Hu Y, Metzler B, Wick G, Xu Q (1998). Mouse model of venous bypass graft arteriosclerosis. *Am J Pathol*.

[18] Bezhaeva T, Wong C, de Vries MR, van der Veer EP, van Alem CMA, Que I (2017). Deficiency of TLR4 homologue RP105 aggravates outward remodeling in a murine model of arteriovenous fistula failure. *Sci Rep*.

[19] Kim D, Langmead B, Salzberg SL (2015). HISAT: a fast spliced aligner with low memory requirements. *Nat Methods*.

[20] Anders S, Pyl PT, Huber W (2015). HTSeq-a Python framework to work with high-throughput sequencing data. *Bioinformatics*.

[21] Love MI, Huber W, Anders S (2014). Moderated estimation of fold change and dispersion for RNA-seq data with DESeq2. *Genome Biol*.

[22] Huang K, Bao H, Yan ZQ, Wang L, Zhang P, Yao QP (2017). MicroRNA-33 protects against neointimal hyperplasia induced by arterial mechanical stretch in the grafted vein. *Cardiovasc Res*.

[23] Chiu JJ, Chien S (2011). Effects of disturbed flow on vascular endothelium: pathophysiological basis and clinical perspectives. *Physiol Rev*.

[24] Sciarretta S, Forte M, Frati G, Sadoshima J (2018). New Insights Into the Role of mTOR Signaling in the Cardiovascular System. *Circ Res*.

[25] Altuvia Y, Landgraf P, Lithwick G, Elefant N, Pfeffer S, Aravin A (2005). Clustering and conservation patterns of human microRNAs. *Nucleic Acids Res*.

[26] Cheng J, Du J (2007). Mechanical stretch simulates proliferation of venous smooth muscle cells through activation of the insulin-like growth factor-1 receptor. *Arterioscler Thromb Vasc Biol*.

[27] Predel HG, Yang Z, von Segesser L, Turina M, Bühler FR, Lüscher TF (1992). Implications of pulsatile stretch on growth of saphenous vein and mammary artery smooth muscle. *Lancet*.

[28] Wong AP, Nili N, Strauss BH (2005). *In vitro* differences between venous and arterial-derived smooth muscle cells: potential modulatory role of decorin. *Cardiovasc Res*.

[29] Zhang WD, Bai HZ, Sawa Y, Yamakawa T, Kadoba K, Taniguchi K (1999). Association of smooth muscle cell phenotypic modulation with extracellular matrix alterations during neointima formation in rabbit vein grafts. *J Vasc Surg*.

[30] Jia G, Mitra AK, Gangahar DM, Agrawal DK (2009). Regulation of cell cycle entry by PTEN in smooth muscle cell proliferation of human coronary artery bypass conduits. *J Cell Mol Med*.

[31] Ishida M, Komori K, Yonemitsu Y, Taguchi K, Onohara T, Sugimachi K (2001). Immunohistochemical phenotypic alterations of rabbit autologous vein grafts implanted under arterial circulation with or without poor distal runoff-implications of vein graft remodeling. *Atherosclerosis*.

[32] Jain M, Dhanesha N, Doddapattar P, Chorawala MR, Nayak MK, Cornelissen A (2020). Smooth muscle cell-specific fibronectin-EDA mediates phenotypic switching and neointimal hyperplasia. *J Clin Invest*.

[33] Nada S, Hondo A, Kasai A, Koike M, Saito K, Uchiyama Y (2009). The novel lipid raft adaptor p18 controls endosome dynamics by anchoring the MEK-ERK pathway to late endosomes. *EMBO J*.

[34] Hosokawa T, Kimura T, Nada S, Okuno T, Ito D, Kang S (2017). Lamtor1 Is Critically Required for CD4^+^ T Cell Proliferation and Regulatory T Cell Suppressive Function. *J Immunol*.

[35] Mori S, Nada S, Kimura H, Tajima S, Takahashi Y, Kitamura A (2014). The mTOR pathway controls cell proliferation by regulating the FoxO3a transcription factor via SGK1 kinase. *PLoS One*.

[36] Halka AT, Turner NJ, Carter A, Ghosh J, Murphy MO, Kirton JP (2008). The effects of stretch on vascular smooth muscle cell phenotype *in vitro*. *Cardiovasc Pathol*.

[37] Jeck WR, Sorrentino JA, Wang K, Slevin MK, Burd CE, Liu J (2013). Circular RNAs are abundant, conserved, and associated with ALU repeats. *RNA*.

[38] Chen LL (2016). The biogenesis and emerging roles of circular RNAs. *Nat Rev Mol Cell Biol*.

[39] Holdt LM, Stahringer A, Sass K, Pichler G, Kulak NA, Wilfert W (2016). Circular non-coding RNA ANRIL modulates ribosomal RNA maturation and atherosclerosis in humans. *Nat Commun*.

[40] Tan WL, Lim BT, Anene-Nzelu CG, Ackers-Johnson M, Dashi A, See K (2017). A landscape of circular RNA expression in the human heart. *Cardiovasc Res*.

[41] Lim TB, Aliwarga E, Luu TDA, Li YP, Ng SL, Annadoray L (2019). Targeting the highly abundant circular RNA circSlc8a1 in cardiomyocytes attenuates pressure overload induced hypertrophy. *Cardiovasc Res*.

[42] Wang YX, Wang DY, Guo YC, Guo J (2019). Zyxin: a mechanotransductor to regulate gene expression. *Eur Rev Med Pharmacol Sci*.

[43] Lützenberg R, Solano K, Buken C (2018). Pathway Analysis Hints Towards Beneficial Effects of Long-Term Vibration on Human Chondrocytes. *Cell Physiol Biochem*.

[44] Terzidou V, Sooranna SR, Kim LU, Thornton S, Bennett PR, Johnson MR (2005). Mechanical stretch up-regulates the human oxytocin receptor in primary human uterine myocytes. J Clin Endocrinol Metab.

[45] Elia L, Quintavalle M, Zhang J, Contu R, Cossu L, Latronico MV (2009). The knockout of miR-143 and -145 alters smooth muscle cell maintenance and vascular homeostasis in mice: correlates with human disease. *Cell Death Differ*.

[46] Yang F, Chen Q, He S, Yang M, Maguire EM, An W (2018). miR-22 Is a Novel Mediator of Vascular Smooth Muscle Cell Phenotypic Modulation and Neointima Formation. *Circulation*.

[47] Ota A, Tagawa H, Karnan S, Tsuzuki S, Karpas A, Kira S (2004). Identification and characterization of a novel gene, C13orf25, as a target for 13q31-q32 amplification in malignant lymphoma. *Cancer Res*.

[48] Ventura A, Young AG, Winslow MM, Lintault L, Meissner A, Erkeland SJ Targeted deletion reveals essential and overlapping functions of the miR-17 through 92 family of miRNA clusters. *Cell*. 200; 132(5):875-86.

[49] He L, He X, Lim LP, de Stanchina E, Xuan Z, Liang Y (2007). A microRNA component of the p53 tumour suppressor network. *Nature*.

[50] Xiao C, Srinivasan L, Calado DP, Patterson HC, Zhang B, Wang J (2008). Lymphoproliferative disease and autoimmunity in mice with increased miR-17-92 expression in lymphocytes. N*at Immunol*.

[51] Mestdagh P, Boström AK, Impens F, Fredlund E, Van Peer G, De Antonellis P (2010). The miR-17-92 microRNA cluster regulates multiple components of the TGF-β pathway in neuroblastoma. *Mol Cell*.

[52] Fiedler J, Thum T (2016). New Insights Into miR-17-92 Cluster Regulation and Angiogenesis. *Circ Res*.

[53] Chen J, Huang ZP, Seok HY, Ding J, Kataoka M, Zhang Z (2013). mir-17-92 cluster is required for and sufficient to induce cardiomyocyte proliferation in postnatal and adult hearts. *Circ Res*.

[54] Wu W, Xiao H, Laguna-Fernandez A, Villarreal G Jr, Wang KC, Geary GG (2011). Flow-Dependent Regulation of Kruppel-Like Factor 2 Is Mediated by MicroRNA-92a. *Circulation*.

