# Supplementary material for: Arterial cyclic stretch regulates Lamtor1 and promotes neointimal hyperplasia via circSlc8a1/miR-20a-5p axis in vein grafts
Source: Theranostics. 2022 Jun 21;12(11):4851–65. doi: 10.7150/thno.69551 (PMC9274756; doi:10.7150/thno.69551)
Supplement: Supplementary file 1 — Supplementary methods, figures and tables. [file thnov12p4851s1.pdf]

Supplemental Materials for

Arterial cyclic stretch regulates Lamtor1 and promotes neointimal hyperplasia via circSlc8a1/miR-20a-5p axis in vein grafts

Ji-Ting Liu[1], Qing-Ping Yao[1], Yi Chen[1], Fan Lv[2], Ze Liu[1], Han Bao[1], Yue Han[1], Ming-Liang Zhang[3]*, Zong-Lai Jiang[1]*, and Ying-Xin Qi[1]*

Supplemental Methods

H&E, Elastin Van-Gieson and Immunofluorescence staining. Grafted veins were fixed in 4% paraformaldehyde, dehydrated in 30% sucrose, and 6 μm cross sections were prepared (LEICA, RM2265). For Hematoxylin/Eosin staining (H&E), sections were stained with Hematoxylin for 4 min, and with Eosin-Y for 30 sec. For Elastin Van-Gieson staining, sections were stained with Weigert Solution for 5 min, directly immerged into Differentiation Solution (1% hydrochloric acid alcohol), and then flushed with water. Van Gieson Dye Solution was used to re-stain the sections for 5-6 min. A microscope (Olympus IX71) was used to observe images. Image J software was used to quantify the area of neointimal hyperplasia which was represented as the pixel point.

For immunofluorescent staining, sections or cells grown on glass coverslips were fixed with 4% paraformaldehyde for 15 min, followed by permeabilization with 0.2% Triton X-100 for 10 min and incubation with blocking buffer containing 10% goat serum for 1 h at room temperature. Antibody against Lamtor1 (CST, 1:200) or phosphor-p70S6K (Thr389) (CST, 1:200) was diluted

in blocking buffer and incubated overnight at 4 °C. Sections or coverslips were washed with PBS 3 times and incubated with secondary antibody (Alexa Fluor 568-conjugated goat anti-rabbit IgG, 1:1000 dilution, Invitrogen) and α-smooth muscle actin-FITC antibody (Sigma, 1:500) for 2 h at room temperature. Nuclei were stained with 4, 6-diamidino-2-phenylindole (DAPI) for 10 min. The images were obtained using a confocal laser scanning microscope (Fluoview 1000, Olympus). All images were quantified with Image J, and the immunofluorescence intensity value of each image was divided by the area of immunostaining region to obtain the average fluorescence intensity.

For each data, 3 independent biological repeats, each with one respective image, were analyzed.

Fluorescence in Situ Hybridization (FISH). Sections were permeabilized with 0.3% Triton X-100 for 30 min at room temperature and washed 3 times with RNase-Free PBS. Sections were treated with Proteinase K (5 μg/mL) for 5 min at 37 °C and then washed with RNase-Free PBS. Specific biotinylated circSlc8a1 probe or scrambled RNA probe was hybridized at 55 °C overnight. Probe detection was performed using Tyramide SuperBoost^{TM} Kit with Alexa Fluor^{TM} Tyramides (TSATM) signal Amplification kit (Invitrogen, B40933) following the manufacturer's instructions. Briefly, samples were incubated at 4 °C overnight in streptavidin-HRP and 3 h in tyramide solution. Nuclei were stained with DAPI and cytoskeleton was stained with α-smooth muscle actin-FITC antibody (sigma, 1:500). The images were obtained using a confocal laser scanning microscope (Fluoview 1000, Olympus), and the immunofluorescence intensity was analyzed with Image J. The sequences of specific circSlc8a1probe and scrambled control probe were list in Table S1.

Cell proliferation assay. Cell proliferation *in vitro* was analyzed using a BrdU ELISA kit (Roche Diagnostics). Eight hours before the end of cyclic stretch application, BrdU labeling reagent was added into the culture medium (1:1000). After stretch application, venous SMCs were seeded with a density of 1.0×10^4 per well in 96-well plates and were labeled according to the manufacturer's instructions. In brief, the cells were fixed with FixDenat solution for 30 min at room temperature and then were incubated with anti-BrdU peroxidase working solution (freshly diluted 1:100) for 90 min. After 3 rinses with washing buffer, substrate solution (100 μL per well) was added onto the cells and incubated for 20 min at room temperature. Thereafter, 25 μL of 1 M H_2SO_4 was added to each well, and the 96-well plates were shaken briefly at 300 rounds per minute. The absorbance at 450 nm was measured in an ELISA plate reader (Bio-Rad 680).

Western Blot. Cellular and tissue proteins were extracted using RIPA lysate buffer containing 1 mM PMSF, and the lysates were centrifugation at 12000 g for 5 min. Cell lysates were separated by SDS-PAGE and then transferred to polyvinylidene fluoride (PVDF) membranes. After blocked with TBST containing 5% no-fat milk for 1 h at room temperature, the membrane was then incubated with following primary antibodies overnight at 4 °C: Lamtor1 rabbit monoclonal antibody (CST, 1:1000), mTORC1 rabbit monoclonal antibody (CST, 1:1000), phosphor-mTORC1 (Ser2448) rabbit monoclonal antibody (CST, 1:1000), phosphor-p70S6K (Thr389) rabbit monoclonal antibody (CST, 1:1000), phosphor-4E-BP1 (Thr37/46) rabbit monoclonal antibody (CST, 1:1000), α-smooth muscle actin rabbit monoclonal antibody (Proteintech, 1:1000),

SM22 rabbit polyclonal antibody (Proteintech, 1:1000), calponin mouse polyclonal antibody (Sigma, 1:1000), and GAPDH mouse monoclonal antibody (Proteintech, 1:5000). Protein bands were visualized by ECL kit (Beyotime) and the intensity was quantified by Quantity One (Bio-Rad). The gray values of mTORC1, p-mTORC1, p-4EBP1, p-p70S6K, Lamtor1, SMA, Calponin and SM22 were compared with that of GAPDH, respectively. Then the protein expression of respective molecule in experimental group was normalized to that of the control.

RNA isolation and quantitative real-time PCR. Total RNAs were isolated using the TRIzol reagent (Invitrogen) according to the manufacturer's protocol. For detection of mRNA, cDNA was reverse transcribed with SuperScript II reverse transcriptase (Invitrogen) using oligo (dT). Quantitative real-time PCR was performed using SYBR green master mix (Applied Biosystems). Housekeeping gene GAPDH was used as internal control.

For miRNA, reverse transcription and PCR were performed using the Hairpin-it™ miRNA qPCR Quantitation Kit (Gene pharma, Shanghai, China), and U6 was used as internal control.

For circRNA, cDNA was reverse transcribed with SuperScript II reverse transcriptase (Invitrogen) using random primers, and PCR was performed using convergent primers and divergent primers. GAPDH was used as internal control.

All assays were performed on the StepOnePlus™ Real-Time PCR Instrument. Relative gene expression was analyzed with $2^{-\Delta\Delta Ct}$ method. The gene specific primers were listed in Table S7.

miRNA and small interfering RNA transfection. Venous SMCs were transfected with mimics,

inhibitor or small interfering RNA (siRNAs) by using Lipofectamine 2000 (Invitrogen) according to the manufacturer's protocol.

For the overexpression or knockdown of miRNAs, venous SMCs were transfected with mimics or inhibitor of miR-17-5p, miR-20a-5p, miR-29a-3p, miR-29c-3p and the corresponding nonsilencing controls (Shanghai GenePharma) respectively. Then cells were harvested for further study after 48 h.

Specific siRNA targeting back-splicing region of circSlc8a1 and specific siRNA targeting *Lamtor1* were designed and synthesized by GenePharma. Nonsilencing siRNA was used as a mock control. Cells were harvested for further study after 48 h.

For circSlc8a1 siRNA treatment *in vivo*, circSlc8a1-specific siRNA or the corresponding nonsilencing siRNA control (Shanghai GenePharma Co., Ltd.) was subcutaneously injected after vein graft *in vivo*. For the first injection, 20 nmol circSlc8a1-specific siRNA or nonsilencing control was subcutaneously injected around the grafted veins, and then, 15 nmol was administered every day for 1 wk.

All the sequences of mimics, inhibitor, and siRNAs were listed in Tables S8, S9 and S10.

RNase R digestion. Total RNA was extracted using TRIzol reagent (Invitrogen). RNase R treatment was performed at 37 °C for 15 min with 3 U RNase R (Epicedtre, Madison, WI). The reaction was stopped at 70 °C for 10 min. For linear mRNA detection, the treated RNA was reverse transcribed using the SuperScript II reverse transcriptase (Invitrogen) with oligo (dT); for circRNA detection, random primers were used. The PCR reactions were conducted using a SYBR green

master mix (Applied Biosystems).

Supplemental Figures

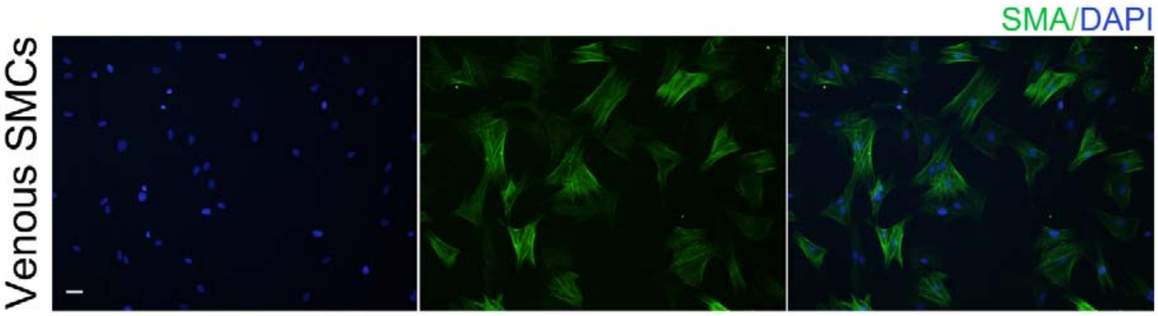

Figure S1. Characterization of isolated venous SMCs. Representative images of isolated venous SMCs from jugular vein stained with α-SMA (green). Nuclei were counterstained with DAPI. Scale bars: 20 μm.

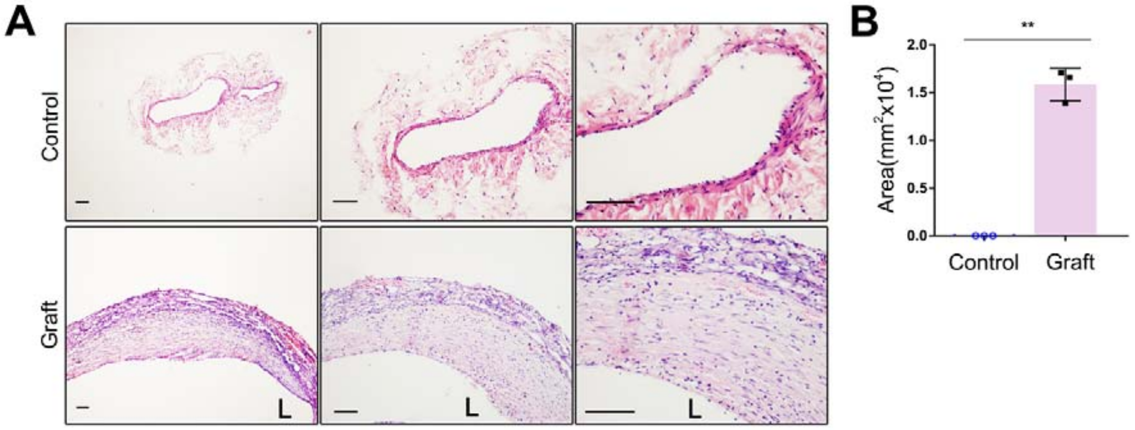

Figure S2. The intimal hyperplasia was significantly increased in rat grafted vein. (A) Representative images of H&E staining showed the vascular wall of grafted vein and contralateral jugular vein (control). L indicated the vessel lumen. Scale bars: 100 μm. (B) Quantification showed the intima area. Each dot represents a single rat (*n* = 3). Values were represented as mean ± *SD*. $^{**}P < 0.01$.

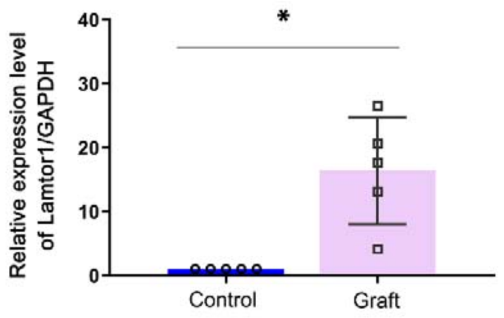

Figure S3. mRNA level of *Lamtor1* was increased in rat grafted vein. Bar graph represented mRNA expression of Lamtor1 normalized to GAPDH which expressed as fold change (n = 5). Values were represented as mean ± *SD*. *P < 0.05.

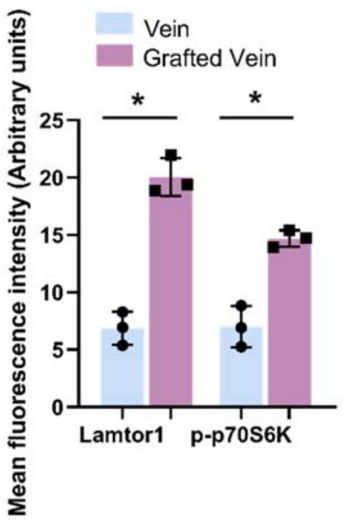

Figure S4. Lamtor1 and p-p70SK qualification of Figure 1B in the manuscript. Each dot represents a single mouse (n = 3). Values were represented as mean ± *SD*. *P < 0.05.

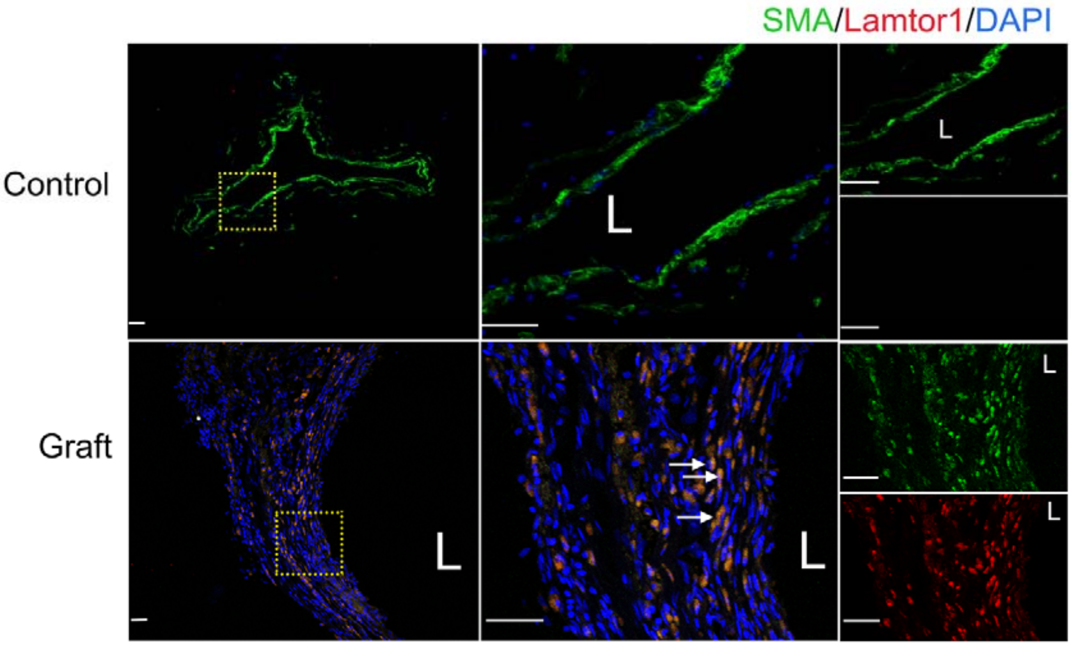

Figure S5. Expression of Lamtor1 was increased in rat intimal hyperplasia after vein graft. Representative images showed double-immunostaining of Lamtor1 (red) and α-smooth muscle actin (α-SMA; green) in grafted vein and contralateral jugular vein (control). Nuclei were counterstained with DAPI (blue). Boxed region was magnified on the right. Arrows represented the co-locolization of α-SMA and Lamtor1. L indicated the vessel lumen. Scale bar: 20 µm.

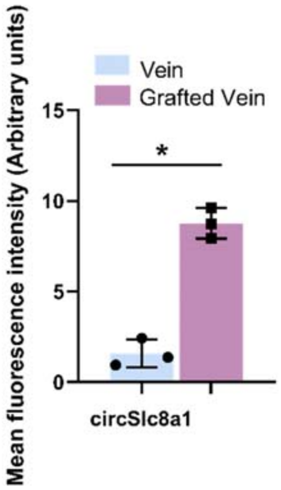

Figure S6. circSlc8a1 qualification of Figure 2G in the manuscript. Each dot represents a single mouse (n = 3). Values were represented as mean ± *SD*. $^{*}P < 0.05$.

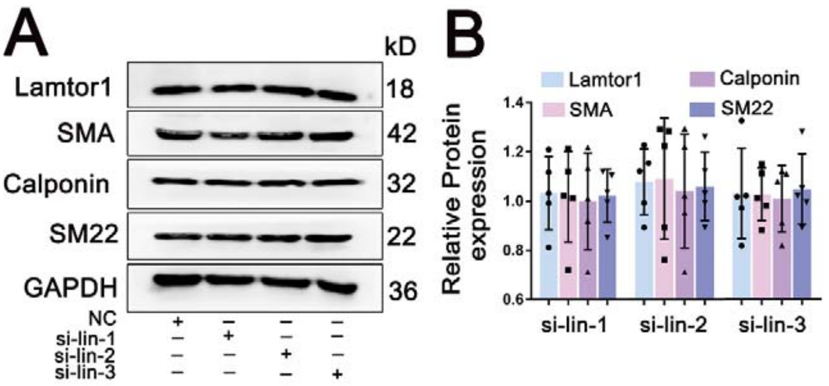

Figure S7. Linear *Slc8a1* had no significant effect on Lamtor1 expression and SMC differentiation. (A) After transfected with 3 kinds of specific siRNA targeting on linear *Slc8a1* (si-lin), the protein levels of Lamtor1 and SMC differentiated markers, including α-SMA, calponin and SM22, were determined by western blot. GAPDH was used as an endogenous control. (B) Densitometric analysis of protein expression normalized to GAPDH (n = 5). Values were represented as mean ± *SD*.

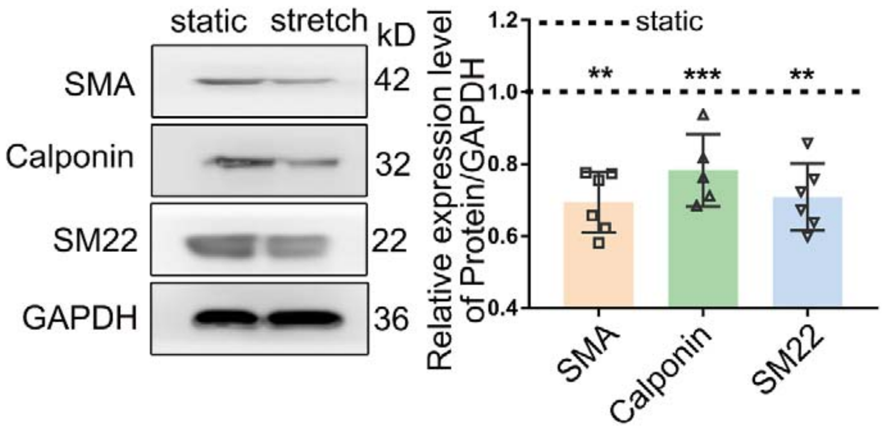

Figure S8. The expressions of differentiation makers were repressed in venous SMCs subjected to 10% cyclic stretch (n = 5). Values were represented as mean ± *SD*. **P < 0.01, ***P < 0.001.

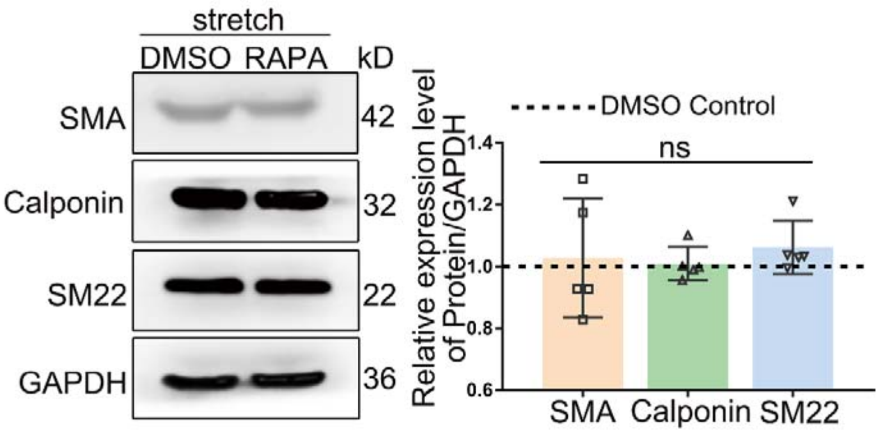

Figure S9. The expressions of differentiation makers were not changed in venous SMCs treated with rapamycin (n = 5). Values are represented as mean ± *SD*.

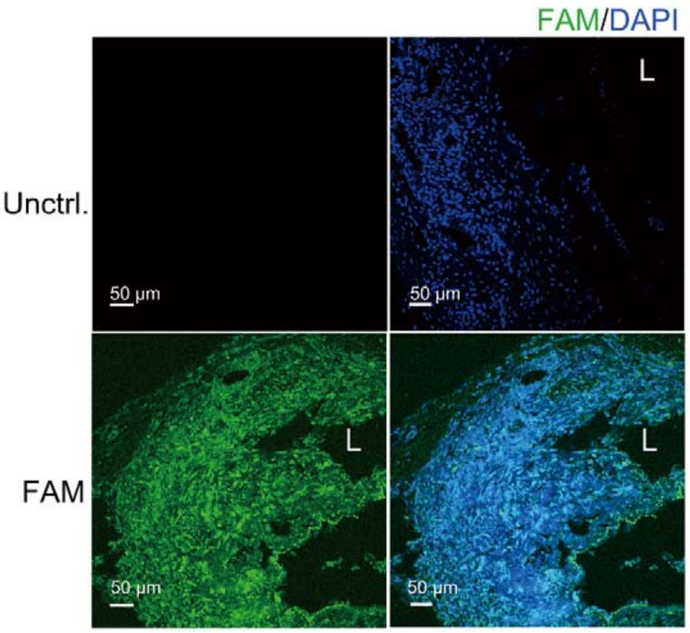

Figure S10. Grafted vein was transfected with FAM labeled siRNA. Representative images showed the siRNA labeled with FAM (green) in grafted vein compared with the unlabeled control (Unctrl.). Nuclei were counterstained with DAPI (blue). L indicated the vessel lumen. Scale bar: 50μm.

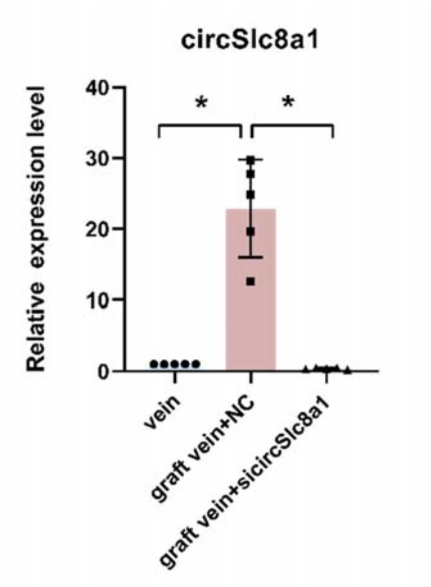

Figure S11. RT-qPCR detected the expression of circSlc8a1 in grafted vein after circSlc8a1-specific siRNA (sicircSlc8a1) treatment for 1 wk compared with the nonsilencing control (NC) (n = 5). Data were represented as mean ± SD. *$P < 0.05$.

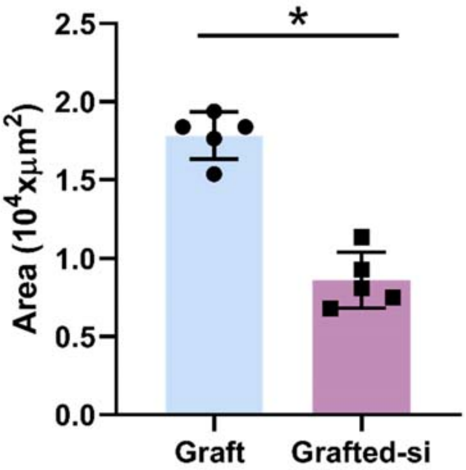

Figure S12. Neointima quantification of Fig. 6A in the manuscript. The neointima area of circSlc8a1-specific siRNA treatment for 1 week in grafted vein compared with the nonsilencing control (NC). Each dot represents a single rat ($n = 5$). Values were represented as mean ± SD. *$P < 0.05$.

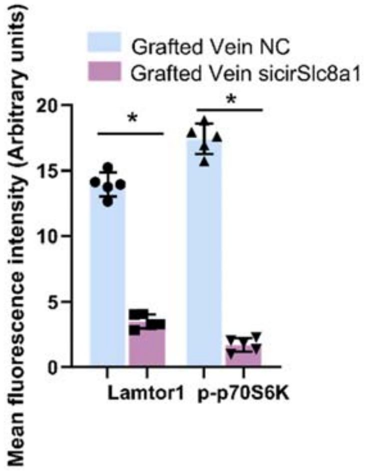

Figure S13. Lamtor1 and p-p70SK qualification of Figure 6C&6D in the manuscript. Each dot represents a single rat (n = 5). Values were represented as mean ± SD. *P < 0.05.

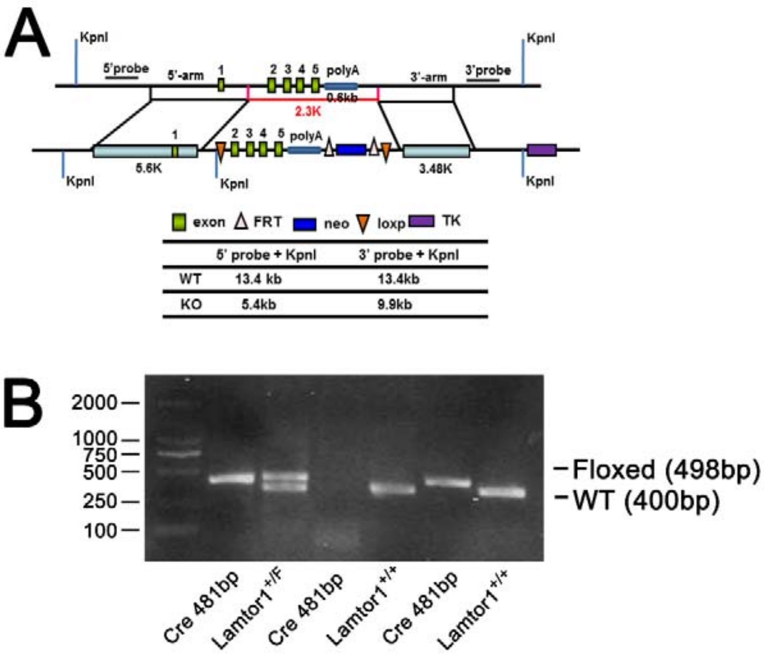

Figure S14. SMC-specific *Lamtor1* knockout (KO) mice. (A) Schematic map showed the strategy to generate SMC-specific *Lamtor1* KO mice. (B) Genomic PCR showed the presence of *SM22*Cre gene in *Lamtor1*$^{fl/fl}$ mice.

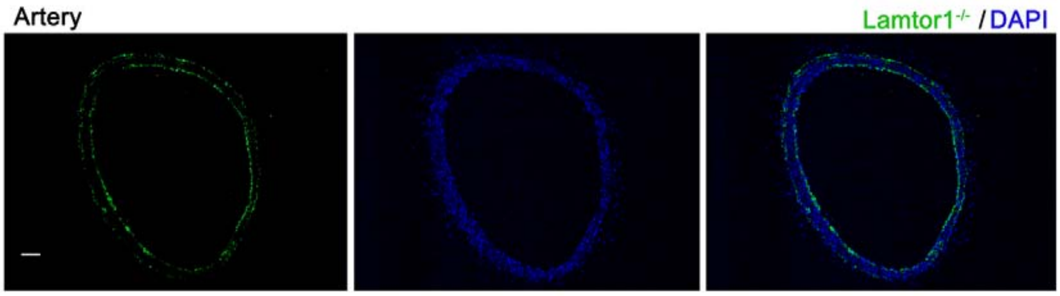

Figure S15. Lamtor1 expression in artery of SMC-specific *Lamtor1* knockout (KO) mice. Representative images showed that there was no immunostaining of Lamtor1 (green) in media of carotid artery. Nuclei are counterstained with DAPI (blue). Scale bar: 50μm.

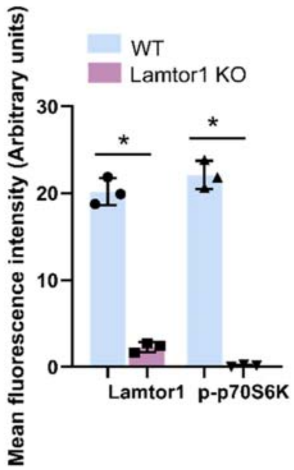

Figure S16. Lamtor1 and p-p70SK qualification of Figure 7A&7D in the manuscript. Each dot represents a single mouse (n = 5). Values were represented as mean ± SD. *$P < 0.05$.

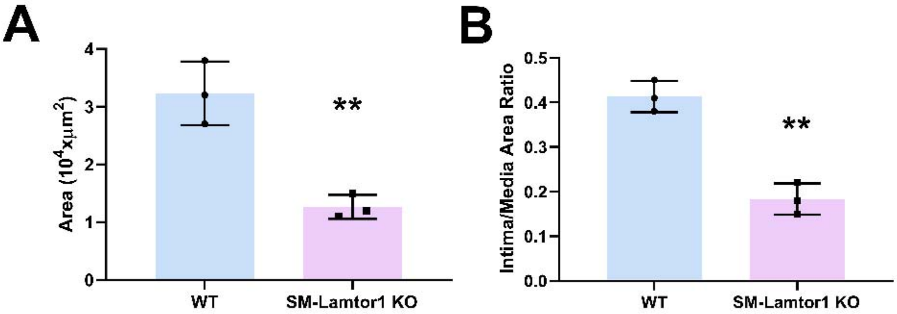

Figure S17. The neointimal area (A) and neointima-to-media ratio (B) of Figure 7B in the manuscript. Each dot represents a single mouse ($n = 3$). Values were represented as mean ± *SD*. $^{**}P < 0.01$.

Supplemental Tables

Table S1. Sequences of probes used in FISH and RNA pull down.

Gene	Sequence of oligonucleotides (5'-3')
circSlc8a1	AGGCTCAGCCCAGGTTGGAACAATT
Scrambled Control	ATGTCGACGAGCACGTATATTA

Table S2. Sequences of circSlc8a1 3'UTR containing the binding site of miR-20a-5p (WT) and the mutant (MUT) for the luciferase reporter assay.

Gene	Sequence
WT	AGAGAATCTAT**gcacttt**CTCTTAAATGCATGACCAGCAGTACAATTAATTTCTGTCTTTTTCCCATGAATTCATTGCATAATAGTTTTGCTTCTAGGGGATGATTAACAAGTTGCTAGTATTATCTGAATACAGATTTTCCTTTATTTTTATTTTATGTGTATGGGTATTTTGCTTAGATGTATGTGTGCACAGTTTATGCACATGTATGTGTGTATAGTCCCCATGAAAGTCAGAAGAGGCCTCAGATTCCAGTAGTTACAGATGGATGCTAGCCTCTTTGTGAGTTATAAAAATCAAGCCTGGGTCCTCTGGAAAAGCAAGAAGAACTCTTAACCACCGATCTATCTCTCCAGTTCTTGAATATAGATTTTAAATCTTGTGATGCAAGTGAAATAAAAAAAGTACATTAGTAAGCTATTTTCTTCTAACTTATTAGCCTT**gcacttt**CTGAAGAAGAGTCAAGGAGATCAAGGAAGG
MUT	AGAGAATCTAT**cgtgaaa**CTCTTAAATGCATGACCAGCAGTACAATTAATTTCTGTCTTTTTCCCATGAATTCATTGCATAATAGTTTTGCTTCTAGGGGATGATTAACAAGTTGCTAGTATTATCTGAATACAGATTTTCCTTTATTTTTATTTTATGTGTATGGGTATTTTGCTTAGATGTATGTGTGCACAGTTTATGCACATGTATGTGTGTATAGTCCCCATGAAAGTCAGAAGAGGCCTCAGATTCCAGTAGTTACAGATGGATGCTAGCCTCTTTGTGAGTTATAAAAATCAAGCCTGGGTCCTCTGGAAAAGCAAGAAGAACTCTTAACCACCGATCTATCTCTCCAGTTCTTGAATATAGATTTTAAATCTTGTGATGCAAGTGAAATAAAAAAAGTACATTAGTAAGCTATTTTCTTCTAACTTATTAGCCTT**cgtgaaa**CTGAAGAAGAGTCAAGGAGATCAAGGAAGG

Table S3. ***Lamtor1*** **expression in RNA-seq data.**

AccID	FoldChange	p-Value	Graft1	Graft2	Graft3	Graft4	Vein1	Vein2	Vein3	Vein4
**Lamtor1**	1.433999	0.048923	284	230	217	227	94	147	158	64

Table S4. The binding ability of circSlc8a1 to miR-17-92 cluster and miR-29 cluster predicted by binding energy.

QueryID	SubjectID	Energy	StartSubject	Endsubject
rno-miR-17-5p	chr6_4520635_3807346_-713289-Slc8a1	-31.8	11309	11329
rno-miR-29a-3p	chr6_4520635_3807346_-713289-Slc8a1	-27	504800	504825
rno-miR-29c-3p	chr6_4520635_3807346_-713289-Slc8a1	-27.4	294413	294438
rno-miR-20a-5p	chr6_4520635_3807346_-713289-Slc8a1	-31.1	11309	11330

Table S5. Potential transcription factors binding with Slc8a1 promoter by JASPAR.

Matrix ID	Name	Score	Sequence ID	Start	End	Predicted sequence
MA1124.1	ZNF24	20.319569	NC_000002.12:c40514435-40512336	1524	1536	CATTCATTCATTA
MA1125.1	ZNF384	16.308338	NC_000002.12:c40514435-40512336	1736	1747	AAAAAAAAAAAA
MA0845.1	FOXB1	16.235079	NC_000002.12:c40514435-40512336	1447	1457	TAAGTAAATAT
MA0032.2	FOXC1	15.998632	NC_000002.12:c40514435-40512336	1447	1457	TAAGTAAATAT
MA0847.3	FOXD2	15.369187	NC_000002.12:c40514435-40512336	1445	1456	GTTAAGTAAATA
MA0508.3	PRDM1	15.319875	NC_000002.12:c40514435-40512336	985	995	CTCTTTCTCTC
MA0803.1	TBX15	15.283173	NC_000002.12:c40514435-40512336	284	291	AGGTGTGA
MA0836.2	CEBPD	15.055553	NC_000002.12:c40514435-40512336	1011	1023	TGTTGCACAAGAT
MA0805.1	TBX1	15.051042	NC_000002.12:c40514435-40512336	284	291	AGGTGTGA
MA1525.2	NFATC4	14.860185	NC_000002.12:c40514435-40512336	953	962	AATGGAAAAT

Table S6. Differentially expressed ncRNAs participated in cellular dedifferentiation based on RNA-seq data and IPA analysis.

Name	p-value range	Molecules
**Cellular dedifferentiation**	5.54E-03-1.11E-07	34
		rno-let-7b-5p, rno-miR-206-3p, miR-1-3p, rno-miR-101a-5p, rno-miR-10b-3p, rno-miR-351-5p, rno-miR-130b-5p, rno-miR-143-3p, rno-miR-145-3p, rno-miR-17-1-3p, rno-miR-20a-5p, rno-miR-18a-5p, rno-miR-19a-5p, rno-miR-205, rno-miR-21-5p, rno-miR-212-5p, rno-miR-223-3p, rno-miR-22-3p, rno-miR-24-1-5p, rno-miR-26a-5p, rno-miR-27b-5p, rno-miR-29c-3p, rno-miR-30a-3p, rno-miR-31a-5p, rno-miR-340-5p, rno-miR-34c-3p, rno-miR-488-3p, rno-miR-503-5p, rno-miR-543-3p, rno-miR-92a-1-5p, rno-miR-99a-3p, LOC100359692, LOC100360950, circSLc8a1

Table S7. Sequences of primers used for quantitative RT-PCR (F: forward; R: reverse).

Gene	Sequence of oligonucleotides (5'-3')
rno-miR-93-5p	F: CGTTATATCCCAAAGTGCTGTTC<br>R: TATGGTTGTTCTCGTCTCCTTCTC
rno-miR-29c-3p	F: CTCCTCCTTTTAGCACCATTTG<br>R: TATGCTTGTTCTCGTCTCTGTGTC
rno-miR-106b-5p	F: AAATGCTCATAAAGTGCTGACAGT<br>R: TATGGTTTTGACGACTGTGTGAT
rno-miR-17-5p	F: ATTCTTCCAAAGTGCTTACAGTGC<br>R: TATGGTTTTGACGACTGTGTGAT
rno-miR-20a-5p	F: GCCGTAAAGTGCTTATAGTGCAG<br>R: TATGGTTTTGACGACTGTGTGAT
rno-miR-20b-5p	F: AGCATACAAAGTGCTCATAGTGC<br>R: TATGGTTTTGACGACTGTGTGAT
rno-miR-29a-3p	F: CATCTGACTAGCACCATCTGAAAT<br>R: TATGGTTTTGACGACTGTGTGAT
rno-miR-29b-3p	F: ACAGCAATTAGCACCATTTGAA<br>R: TATGCTTCTTCTCGTCTCTGTGTC
Lamtor1	F: TCTAGTACCCGCTTGGCTGTGCTTA<br>R: GCTCTTCTTTCGCATCCAC3
SLC8A1	F: GCGGAGGCCAGAAATAGGAG<br>R:ATCCCGAATCCACTCACCTTG
GAPDH	F: TGAACTTGCCGTGGGTAGAG<br>R:GATGGTGAAGGTCGGTGTGA
circPhf11	F: CAATCTTCTCCCTCTCC<br>R:GTCTGCTGTATTCATCA<br>F:ATTGAAACATTGGGTGGGAGAC

circSlc8a1	R:CTCAGCCCAGGTTGGAACAA
circTfap4	F:ACAAGATAGGAGCATTC<br>R:CAGCAGCACCTGACAGA
circPlscr2	F:CACACGGACCAACGACC<br>R:CTGCTGCCTCCAGGAGA
circMyh8	F:TCCTGCTTCTGCTGTCC<br>R:CCTTGGTTTCTTCCTCG

Table S8. Sequences of miRNA mimics used in this study.

Gene	Sequence of oligonucleotides (5'-3')
rno-miR-29b-3p	UAGCACCAUUUGAAAUCAGUGUU<br>CACUGAUUUCAAUGGUGCUAUU
rno-miR-29a-3p	UAGCACCAUCUGAAAUCGGUUA<br>ACCGAUUUCAGAUGGUGCUAUU
rno-miR-20b-5p	CAAAGUGCUCAUAGUGCAGGUAG<br>ACCUGCACUAUGAGCACUUUGUU
rno-miR-20a-5p	UAAAGUGCUUAUAGUGCAGGUAG<br>ACCUGCACUAUAAGCACUUUAUU
rno-miR-17-5p	CAAAGUGCUUACAGUGCAGGUAG<br>ACCUGCACUGUAAGCACUUUGUU
rno-miR-106b-5p	UAAAGUGCUGACAGUGCAGAU<br>CUGCACUGUCAGCACUUUAUU
rno-miR-29c-3p	UAGCACCAUUUGAAAUCGGUUA<br>ACCGAUUUCAAAUGGUGCUAUU
rno-miR-93-5p	CAAAGUGCUGUUCGUGCAGGUAG<br>ACCUGCACGAACAGCACUUUGUU
Negative control	UUCUCCGAACGUGUCACGUTT<br>ACGUGACACGUUCGGAGAATT

Table S9. Sequences of miRNA inhibitors used in this study.

Gene	Sequence of oligonucleotides (5'-3')
rno-miR-29b-3p	AACACUGAUUUCAAAUGGUGCUA
rno-miR-29a-3p	UAACCGAUUUCAGAUGGUGCUA
rno-miR-20b-5p	CUACCUGCACUAUGAGCACUUUG
rno-miR-20a-5p	CUACCUGCACUAUAAGCACUUUA
rno-miR-17-5p	CUACCUGCACUGUAAGCACUUUG
rno-miR-106b-5p	AUCUGCACUGUCAGCACUUUA
rno-miR-29c-3p	UAACCGAUUUCAAAUGGUGCUA
rno-miR-93-5p	CUACCUGCACGAACAGCACUUUG
Negative control	CAGUACUUUUGUGUAGUACAA

Table S10. Sequences of siRNAs used in this study.

Gene	Sequence of oligonucleotides (5'-3')
Lamtor1-Rat-165	GCCGAGCCCAGCUACCAUATT
	UAUGGUAGCUGGGCUCGGCTT
Lamtor1-Rat-235	CCAAGACAGCUAGCAACAUTT
	AUGUUGCUAGCUGUCUUGGTT
Lamtor1-Rat-346	GCAGCAGUCUGACCCAUUGTT
	CAAUGGGUCAGACUGCUGCTT
Negative control	UUCUCCGAACGUGUCACGUTT
	ACGUGACACGUUCGGAGAATT
circSlc8a1-1	CAACCUGGGCUGAGCCUUGTT
	CAAGGCUCAGCCCAGGUUGTT
circSlc8a1-2	UUCCAACCUGGGCUGAGCCTT
	GGCUCAGCCCAGGUUGGAATT
SLC8A1-1301	GCCCUCACCAUUAUUCGAATT
	UUCGAAUAAUGGUGAGGGCTT
SLC8A1-3068	GGUUCUAGAUGUAGUCUAATT
	UUAGACUACAUCUAGAACCTT
SLC8A1-1027	GCAGAAGCAUCCCGACAAATT
	UUUGUCGGGAUGCUUCUGCTT
